# Identification and Comparison of Potential Biomarkers by Proteomic Analysis in Traditional Chinese Medicine-Based Heart Failure Syndromes

**DOI:** 10.1155/2022/6338508

**Published:** 2022-01-18

**Authors:** Yang Jiang, Qi Zhao, Shumin Huang, Bin Cheng, Zhixi Hu

**Affiliations:** ^1^College of Traditional Chinese Medicine, Hunan University of Chinese Medicine, Changsha 410208, China; ^2^Hunan Academy of Traditional Chinese Medicine Affiliated Hospital, Changsha 410006, China

## Abstract

Heart failure (HF) is an epidemic disease affecting a large population worldwide. Traditional Chinese medicine (TCM) is playing an increasingly important role in the clinical treatment of HF. According to the TCM theory, HF could be classified into Yang deficiency and Qi-yin deficiency; however, there are few objective and biological lines of evidence for differentiation of TCM HF syndromes to date. In this study, data-independent acquisition (DIA) mass spectrometry was applied to comparatively analyze the protein expression in serum samples obtained from 12 Yang deficiency patients, 12 Qi-yin deficiency patients, and 12 healthy volunteers. Compared to the healthy controls, a total of 121 differentially expressed proteins (DEPs) (77 upregulated and 44 downregulated proteins) were identified in Yang deficiency samples, while 59 DEPs (49 upregulated and 10 downregulated proteins) were detected in Qi-yin deficiency samples. Enrichment analyses of these DEPs based on the GO and KEGG databases revealed functional clusters associated with the immune system, signal transduction, and infectious disease. Several previously reported HF biomarker proteins were found to be the hub proteins in a protein-protein interaction network analysis. Three novel hub DEPs were identified as potential biomarkers for differentiation between different TCM syndromes of HF. The results provide biological insight into the differences of different TCM HF syndromes and an opportunity for specific biomarker identification for different TCM HF syndromes.

## 1. Introduction

Heart failure (HF) is a progressive and complex clinical syndrome characterized by symptoms such as breathlessness, ankle swelling, and fatigue and is associated with high morbidity and high mortality. HF is the end stage of various cardiac diseases, such as valvular heart disease, coronary artery disease, and hypertension, which primarily have myocardial damage in common as pathogenesis. In developed countries, the prevalence of HF is approximately 1%-2% in the adult population, while it is ≥10% among people above 70 years of age [1]. Due to the rapidly aging global population, the worldwide prevalence of HF is expected to continuously increase [2].

In modern Western medicine practice, HF patients are mainly treated with angiotensin-converting enzyme inhibitors, cardiotonic drugs, and diuretics [3]. In China, it is common for these patients to receive a combination of Western medicine and traditional Chinese medicine (TCM). TCM has become an important complementary and alternative medical approach, not only widely adopted in China but also becoming increasingly prevalent and accepted globally. In recent years, this type of integrated treatment of HF has shown significant progress and effectiveness. It has been reported to improve heart function and symptoms such as fatigue and dyspnea and enhance the survival time of patients and their quality of life [4–7].

In TCM practice, the diagnosis and treatment of diseases are based on syndrome differentiation (defined as Bian Zheng) and mainly depend on observation, listening, questioning, and pulse analysis [8]. According to the “Consensus of TCM and TCM and Western Medicine Diagnostics Experts on Chronic Heart Failure,” HF patients can be classified into three subtypes: Qi deficiency syndrome, Yang deficiency syndrome, and Yin deficiency syndrome [9]. In addition, the Qi deficiency syndrome and Yin deficiency syndrome could be combined together and defined as Qi-yin deficiency syndrome. Since TCM herbal formulae are prescribed individually based on the differentiation of TCM syndromes, accurate differentiation is vital and is the key principle of effective TCM treatments [8]. However, unlike conventional medicine which uses objective laboratory tests and imaging techniques as diagnostic tools, syndrome classification in TCM is solely based on knowledge, observation, and clinical experience of TCM practitioners. Because these methods lack objectivity, repeatability, and standard guidelines, they have drawn skepticism and criticism [10]. Therefore, a scientific and objective approach to TCM syndrome differentiation is urgently needed.

Since a decade, systems biology technology, in particular proteomics, has been increasingly adopted in TCM research [10–13]. The development and pathophysiology of HF are associated with changes in the amount of certain serum proteins [14]. These results could help us understand the corresponding pathology and uncover potential biomarkers for TCM diagnoses. The relationships of these proteins with HF allow physicians to assess the presence, severity, and/or prognosis with increased precision and accuracy. The clinical diagnosis and management of HF in conventional medicine are facilitated by several protein-based circulating biomarkers like brain natriuretic peptide (BNP) [15, 16] and ST2 [17, 18]. Currently, no specific biomarker has been reported in TCM diagnosis of different syndrome types of HF. Extensive research is still needed to isolate and identify novel protein biomarkers associated with HF syndromes [19]. The discovery of objective and reliable biomarkers that can be used in the differentiation of HF syndromes in TCM has the potential to significantly improve the accuracy of disease assessment and management and uncover biological insights which could lead to novel treatments [20]. Due to the high sensitivity, accuracy, and throughput, data-independent acquisition- (DIA-) based quantitative proteomic analysis has been widely used for identification of disease biomarkers in different types of diseases [20–22]. In the present study, we aimed to identify the serum protein biomarkers of Yang deficiency and Qi-yin deficiency syndromes in HF patients by employing proteomics technology coupled with bioinformatics analyses. Differentially expressed proteins (DEPs) of HF patients with Yang deficiency or Qi-yin deficiency syndromes as well as healthy people were first identified with DIA-based mass spectrometry (MS) and then compared. Subsequently, further comparative analysis of the DEPs was performed by Gene Ontology (GO), Kyoto Encyclopedia of Genes and Genomes (KEGG) pathways, and protein-protein interaction (PPI) networks to gain insight on the biological differences between the Yang deficiency and Qi-yin deficiency TCM syndromes of HF.

## 2. Materials and Methods

### 2.1. Ethics Statement

This study was performed in accordance with the Declaration of Helsinki. The use of human serum samples was reviewed and approved by the research medical ethics committee of Hunan Academy of Traditional Chinese Medicine Affiliated Hospital (registration number: ChiCTR2000033155), and signed informed consent was obtained from all participants prior to enrollment.

### 2.2. Patients

A total of 24 treatment-naïve HF patients (12 Yang deficiency syndrome and 12 Qi-yin deficiency syndrome) and 12 healthy controls were enrolled from the Department of Cardiology of Hunan Academy of Traditional Chinese Medicine Affiliated Hospital (China) between June 2020 and December 2020. Clinical data of the enrolled participants are shown in Table 1. The distributions of age and gender between different groups showed no statistical difference (*P* > 0.05).

All the participants were diagnosed by chief physicians according to both western medicine [3] and TCM criteria for syndrome differentiation [8, 9]. According to *expert consensus on TCM treatment of chronic heart failure*, Yang deficiency syndrome is mainly defined by shortness of breath or wheezing, fatigue, and palpitation. Secondary symptoms include being afraid of cold and (or) preference for warmth, cold sensation of abdomen, waist, or limbs, cold sweat, complexion, dark purple lips, dark purple tongue (or with ecchymosis or purplish veins under the tongue), fat and large tongue with possible tooth marks, and a thin, deep, and weak pulse. Meanwhile Qi-yin deficiency syndrome is defined by palpitation, shortness of breath, tiredness, fatigue, nighttime perspiration, dry mouth, dark red zygoma, red tongue with little coating, and a thready and rapid pulse or irregular intermittent pulse. The following exclusion criteria were applied: (a) Patients who did not sign the informed consent or participated in other studies in the past month; (b) diagnosed with other severe primary disease(s) such as severe liver, kidney, hematopoietic system diseases, and/or malignant tumors; (c) patients with acute coronary syndrome, pulmonary embolism, or acute cerebrovascular disease; (d) patients with a history of organ transplantation or psychosis with poor control; (e) drug addicts without detoxification; and (f) age <18 or >85 years.

### 2.3. Sample Collection

After thorough clinical evaluation, patients were excluded according to the criteria stated above. For proteomics analysis, blood samples of 12 Yang deficiency patients, 12 Qi-yin deficiency patients, and 12 healthy controls were obtained. The peripheral blood samples (4 mL) were collected from HF patients and controls, following fasting overnight, without anticoagulation. Within 1 hr after collection, the samples were centrifuged at 3500 rpm for 10 min at 4°C. All the plasma samples were stored at −80°C for further analysis.

### 2.4. Protein Preparation and Extraction

A total of 36 serum samples were obtained and screened. To reduce individual differences and biased parameter estimation, the 12 serum samples per group (Yang deficiency, Qi-yin deficiency, and healthy controls) were divided into four subgroups. This resulted in three samples in each subgroup, which were pooled. Thus, there were four biological replicates for each clinical phenotype and 12 in total for analysis. Then 900 *μ*L SDS-free L3 lysate solution was added to 100 *μ*L of serum sample. The following was conducted to reduce and alkylate the proteins in order to disrupt the disulfide bonds: addition of 10 mM dithiothreitol (DTT) to the samples and incubation for 30 min at 37°C; then, the samples were alkylated with 55 mM iodoacetamide and incubated in the dark for 30 min at room temperature; and lastly, after the treatments, the proteins were enriched using a Gemini C18 column for liquid phase separation of the samples. Protein concentration was quantified using Bradford Protein Assay Kit (CWBIO, Beijing, China) and the prepared proteins were stored at −80°C for subsequent analysis.

### 2.5. Peptide Separation

The same amount of peptides was taken from each sample and mixed equally (10 *μ*g/sample), followed by dilution of 20 *μ*g mixture with 2 mL of buffer A (5% ACN, pH 9.8), which was injected into an LC-20AB liquid phase system. Subsequently, a Gemini C18 column (5 *μ*m, 4.6 × 250 mm) was used for liquid phase separation. Gradient elution was applied to the samples at a flow rate of 4 *μ*L/min in the following gradients: 2% buffer B (95% ACN, pH 9.8) for 7 min, 2% to 7% buffer B for 3 min, 7% to 25% buffer B for 27 min, 25% to 60% buffer B for 2 min, 60% to 80% buffer B for 1 min, with buffer B held for 3 min, and 80% to 2% buffer B for 2 min, and 5% buffer B was equilibrated for 5 min. The elution peaks were observed at a wavelength of 214 nm, while one fraction was obtained every 3.15 min. The samples were mixed with the elution peaks of the chromatogram to collect 10 fractions in total and then freeze-dried.

### 2.6. Data-Dependent and Data-Independent Acquisition Analysis

The dried peptide samples were redissolved in buffer A (2% ACN, 0.1% FA) and centrifuged for 10 min at 20,000 g, and the supernatant was injected into an UltiMate 3000 UHPLC (Thermo, USA) for separation. The samples were first enriched and desalted in a trap column and then eluted onto a self-packed C18 column (150 *µ*m inner diameter, 1.8 *µ*m column diameter, and 35 cm column length). The peptides were separated at a flow rate of 500 nL/min with the following effective gradient: 0–5 min, 5% buffer B (98% ACN, 0.1% FA); 5–90 min, 5% to 25% buffer B; 90–100 min, 25% to 35% buffer B; 100–108 min, 35% to 80% buffer B; 108–113 min, 80% buffer B; and 113–120 min, 5% buffer B. The end of the nanoliter liquid phase separator was directly linked to the MS.

The peptides, which were separated by liquid phase, were ionized by nanoESI and then injected into the Q-Exactive HF tandem MS (Thermo, USA) for data-dependent acquisition (DDA) and DIA analysis. The main settings of the MS and HCD-MS/MS were as follows for DDA analysis: MS: scan range 350–1,500 (m/z), mass resolution 120,000, and maximum injection time (MIT) 100 ms; HCD-MS/MS: collision energy 28 eV, mass resolution 30,000, MIT 100 ms, and 30 s of dynamic exclusion. For DIA analysis, the parameters were set as follows: (1) MS: scan range 400–1,250 (m/z), mass resolution 120,000, and MIT 50 ms; (2) HCD-MS/MS: isolation window 1.7 m/z, mass resolution 30,000, automatic MIT, and 30 s of dynamic exclusion.

### 2.7. Data Processing and Peptide Identification

Raw mass spectral DDA data were searched against the UniProtKB/Swiss-Prot *H. sapiens* proteome database using MaxQuant software (version 1.5.3.30, Germany). The final spectral library was built with peptide/protein entries that satisfied the false-discovery rate (FDR) of ≤1%. Search parameters in the MaxQuant software were selected as follows: enzyme = Trypsin/P; minimal peptide length = 7; PSM-level FDR and protein FDR = 0.01; fixed modifications: carbamidomethyl (C); and variable modifications: oxidation (M) and acetyl (protein N-term). DIA data were analyzed using Spectronaut, which uses iRT peptides to calibrate the retention time. FDR was estimated using the mProphet scoring algorithm, which accurately reflects the matching degree of ion pairs. Then, based on the target-decoy model applicable to SWATH-MS, the false-positive control was completed with FDR ≤1%. DEPs between the three groups were screened using MSstats and fold change ≥2.0 and *P* < 0.05 were the criteria for determining significant differences.

### 2.8. Bioinformatics Analysis

Gene Ontology (GO) annotation was performed using the UniProt-GOA Database (https://www.ebi.ac.uk/GOA/). The molecular function, biological processes, and cellular components of the identified proteins were analyzed.

Annotation of the protein-signaling pathway was conducted with the Kyoto Encyclopedia of Genes and Genomes (KEGG) database. The KEGG Automatic Annotation Server (KAAS) was first used to annotate the DEPs, followed by mapping the results with the KEGG mapper.

The protein-protein interaction (PPI) network was analyzed using the STRING database, from which interactions with a high confidence score of ≥0.7 were selected.

### 2.9. Statistical Analysis

Statistical analyses were conducted with GraphPad Prism and SPSS software.

Pearson's correlation coefficient was calculated between samples to demonstrate correlation of protein quantification and represented as a heat map. The clinical data were analyzed by one-way ANOVA and expressed as mean ± standard deviation (SD). The degree of GO terms enrichment was analyzed with Fisher's exact test. A *P* value <0.05 was considered as statistically significant.

## 3. Results

### 3.1. Clinical Data

The clinical data of HF patients and the control group are shown in Table 1. Table 1 indicates that the mean ages in the Yang deficiency, Qi-yin deficiency, and control group were 71.8, 76.6, and 67.8 years, respectively. The distributions of age and gender were not significantly different between the three groups (*P* > 0.05). However, hemoglobin, blood platelets, and natrium were significantly lower, while creatinine and BNP were significantly higher in the Yang deficiency and Qi-yin deficiency groups compared to the healthy controls. Liver functions and C-Reactive Protein (CRP) did not show any significant differences between the groups.

### 3.2. Quantification of Serum Protein Profiles

The serum samples of Yang deficiency, Qi-yin deficiency, and healthy controls were analyzed by DDA-based MS. A total of 20322 peptides and 2738 proteins were obtained after applying an FDR of ≤1%.

**Figure 1:**
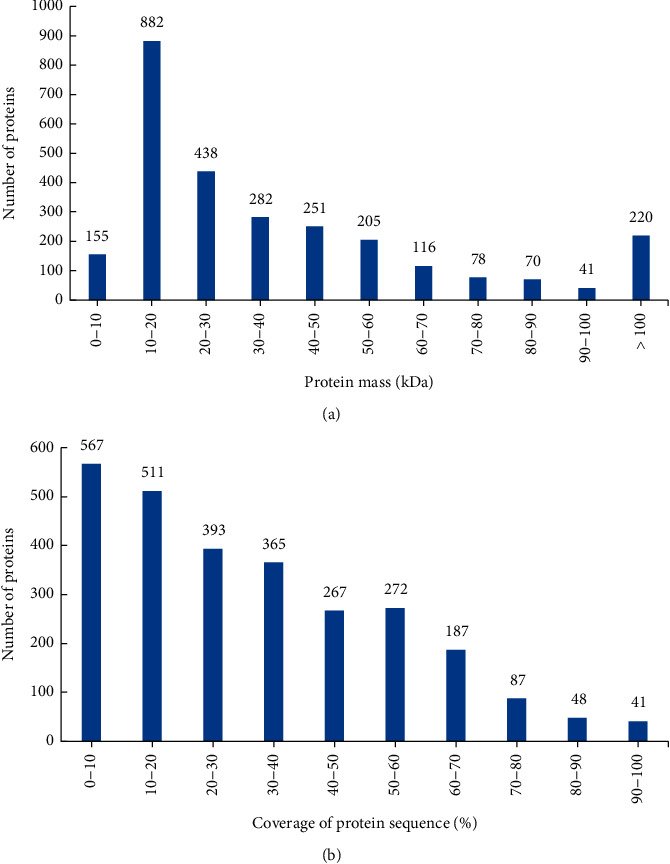
Identification and analysis of the serum samples of heart failure patients. (a) Protein mass distribution of identified proteins. (b) Coverage of identified proteins.

The mass distribution of the identified proteins (Figure 1(a)) revealed that 2583 (94.34%) of the proteins were above 10 kDa, of which 220 (8.03%) were above 100 kDa. In addition, the coverage of protein sequence under 10% accounted for 20.7% (Figure 1(b)).

### 3.3. Identification of the DEPs in Serum

**Figure 2:**
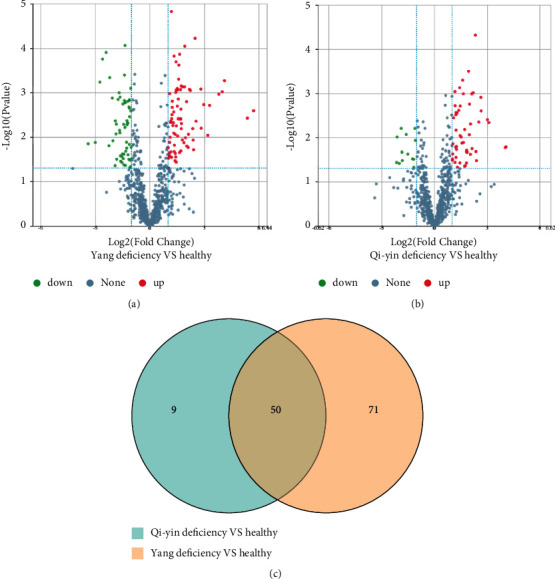
Identification of DEPs in different TCM HF samples. (a, b) DEPs in Yang deficiency and Qi-yin deficiency samples compared to healthy controls. The *X*-axis represents the difference in protein expression level (log2-transformed fold changes), and the *Y*-axis the corresponding log10-transformed *P* values. Red dots indicate significantly upregulated proteins, green dots significantly downregulated proteins, and gray dots symbolize proteins with no significant change. (c) Venn diagram of DEPs in Yang deficiency and Qi-yin deficiency patients.

The identified proteins were quantitively analyzed and those with fold change ≥2 in relative abundance and *P* < 0.05 were identified as DEPs. As shown in the volcano plots, 121 DEPs, 77 upregulated (S1 file) and 44 downregulated proteins (S2 file), were identified in the Yang deficiency group (Figure 2(a)). Meanwhile 59 DEPs, 49 upregulated (S3 file) and 10 downregulated proteins (S4 file), were identified in the Qi-yin deficiency group (Figure 2(b)). The Venn diagram indicated (Figure 2(c)) that 50 DEPs were shared in both the Yang deficiency and Qi-yin deficiency syndromes: 41 were upregulated and 9 downregulated. In addition, there were 71 and 9 DEPs specifically involved in Yang deficiency and Qi-yin deficiency syndromes, respectively.

### 3.4. GO Annotation and Functional Classification of DEPs

**Figure 3:**
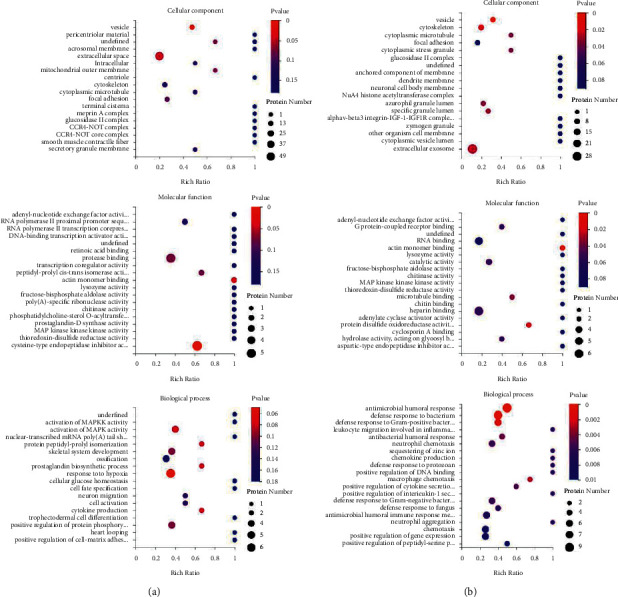
GO annotation of DEPs. (a) Yang-deficiency. (b) Qi-yin deficiency.

To further analyze the biological differences between Yang deficiency and Qi-yin deficiency HF syndromes, GO enrichment analysis was conducted to determine the biological functions of the identified DEPs in the three following categories: cellular components, molecular functions, and biological processes. The results showed that the DEPs were mainly involved in cellular components (Figure 3(a) for Yang deficiency and Figure 3(b) for Qi-yin deficiency). A relatively small number of DEPs were classified into the categories of molecular functions and biological processes (Figures 3(a) and 3(b)). In addition, the DEPs were enriched in syndrome-specific terms. For molecular functions, the DEPs in Yang deficiency were related to protease binding (GO:0002020) and cysteine-type endopeptidase inhibitor activity (GO:0004869). Meanwhile the DEPs in Qi-yin deficiency were mostly enriched in RNA binding (GO:0003723) and heparin binding (GO:0008201). For biological processes, DEPs in Yang deficiency were mainly related to the response to hypoxia (GO:0001666) and activation of MAPK activity (GO:0000187). However, in Qi-yin deficiency, they were mainly related to the antimicrobial humoral response (GO:0019730) and defense response to bacterium (GO:0042742).

**Figure 4:**
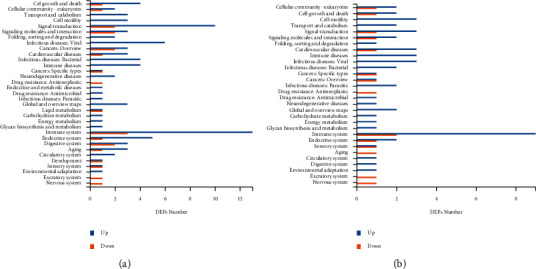
KEGG pathway analyses of the DEPs in Yang deficiency (a) and Qi-yin deficiency (b) groups.

KEGG pathway analysis indicated that the DEPs in Yang deficiency and Qi-yin deficiency samples were both significantly (*P* < 0.05) enriched in immune system (hsa04657), signal transduction (hsa04013, hsa04015, and hsa04391), cardiovascular diseases (hsa05410 and hsa05414), and infectious disease (hsa04114) (Figures 4(a) and 4(b)), indicating that these two TCM HF syndromes were closely related to pathology in immunoreaction.

**Figure 5:**
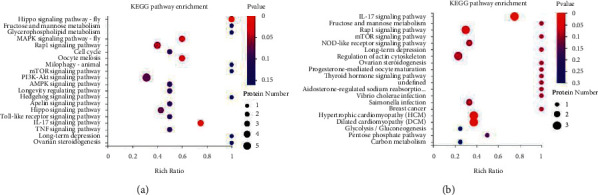
KEGG pathway annotation. (a) Yang deficiency. (b) Qi-yin deficiency.

However, the number of enriched DEPs of each KEGG term showed great differences between Yang deficiency and Qi-yin deficiency syndromes, indicating that these two different types of syndromes have different biological responses (Figures 5(a) and 5(b)). In addition, the term lipid metabolism (hsa00564) was exclusively enriched in Yang deficiency.

### 3.5. PPI Networks of the DEPs

**Figure 6:**
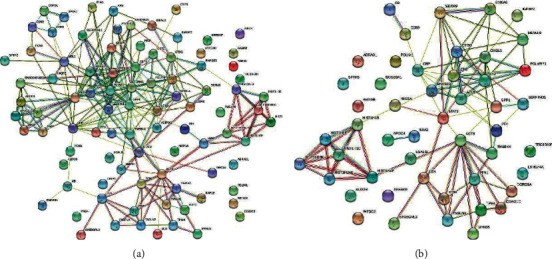
PPI network of the DEPs in (a) Yang deficiency and (b) Qi-yin deficiency groups.

Identifying DEPs in Yang deficiency and Qi-yin deficiency syndromes relative to the control group was of interest as they could give an indication of potentially valuable diagnostic and prognostic biomarkers. In order to identify the hub proteins involved in HF, the PPI networks of the significant DEPs in Yang deficiency and Qi-yin deficiency samples were analyzed by STRING (Figures 6(a) and 6(b)). The results showed that several previously reported biomarkers for HF were specifically related to the PPI network, such as CRP and neutrophil gelatinase-associated lipocalin (LCN2). In addition, several disease related proteins were exclusively involved in the PPI network of the Yang deficiency group (Figure 6(a)), such as adiponectin receptor protein 1 (ADIPOQ), mannose-binding protein C (MBL2), and insulin-like growth factor-binding protein 4 (IGFBP4). Considering the different regulated expression levels, ADIPOQ, MBL2, and IGFBP4 might be potential biomarkers for differentiation and identification of Yang deficiency and Qi-yin deficiency syndromes of HF.

## 4. Discussion

The integration of TCM with conventional Western medicine has been gaining increased attention of the medical community since both adverse reactions to Western medicine in the treatment of HF as well as unique advantages of TCM have been reported.

During the past few decades, the treatment of HF by TCM has made significant progress, especially in improving the clinical symptoms of patients, controlling the development of the disease, and improving the quality of life of patients. The clinical treatment results demonstrated that TCM could relieve the symptoms, improve the quality of life of patients, and prolong the survival time of patients [23–26]. According to the syndrome differentiation in TCM, HF has been divided into Yang deficiency and Qi-yin deficiency syndromes. The following symptoms are associated with Yang deficiency: spontaneous perspiration, aversion to cold and cold limbs, anorexia and abdominal distension, loose stools, soreness and lumbago, dizziness and amnesia, pale complexion, a dark pale tongue or pale and corpulent tongue with teeth prints, and deep, slow, or irregular intermittent pulse. Meanwhile Qi-yin deficiency is associated with the following symptoms: palpitation, shortness of breath, tiredness, fatigue, nighttime perspiration, dry mouth, dark red zygoma, red tongue with little coating, and thready and rapid pulse or irregular intermittent pulse [26]. However, the biological differences between these two types of HF syndromes have not been investigated so far. In the present paper, the proteomic profile of these two types of HF syndromes was analyzed by DIA-based proteomic analysis. The results showed that Yang deficiency and Qi-yin deficiency had a significant difference in number of DEPs compared with the healthy controls (Figure 2). In the serum samples of Yang deficiency patients, 121 DEPs were detected: 77 were upregulated and 44 downregulated. Meanwhile only 59 DEPs were identified in Qi-yin deficiency patients: 49 were upregulated and 10 downregulated.

Although the numbers of DEPs were significantly different between these two types of HF syndromes, 50 common DEPs were also identified as shown in the Venn diagram (Figure 2(c)). The difference in numbers of DEPs was consistent with the different syndromes of these two types of HF. The proteomic profile obtained in this research could help to explain the biological differences in Yang deficiency and Qi-yin deficiency and identify specific biomarkers for syndrome differentiation in clinical diagnosis.

Based on proteomic analysis, previous studies have identified a number of proteins as potential biomarkers for the clinical diagnosis of HF [14, 27], including CRP [28–30], neutrophil gelatinase-associated lipocalin (NGAL) [31, 32], and ST2 [17, 18].

In the present study, we found that several previously reported biomarkers were detected to be upregulated, including CRP, NGAL, and Cystatin C. CRP was first identified as a biomarker of inflammation and high concentrations are associated with mortality in patients with acute myocardial infarction [33]. Other studies later indicated that CRP can be used as a predictor for deterioration of heart function and increased CRP levels could reflect the degree of myocardial damage and showed associations with HF severity, mortality and morbidity, and rehospitalization [28, 30]. Our results indicated that the CRP was significantly upregulated in both Yang deficiency and Qi-yin deficiency patients. However, CRP protein showed a higher induced level in Yang deficiency (upregulated by 15.8-fold) compared to Qi-yin deficiency (upregulated by 8.6-fold) patients (S1 and S3 files). Cystatin C is a small cysteine protease inhibitor protein (120 amino acid peptide chain, approximately 13 kDa) which is synthesized by nucleated cells. Cystatin C is involved in the extracellular inhibition of cathepsin and removed from the circulation by tubular cells in the kidneys, which resulted in the identification of this protein as a biomarker for renal dysfunction and cardiovascular disease [34, 35]. Previous studies have demonstrated that the serum Cystatin C level is associated with HF and could be used as biomarker for cardiac diastolic dysfunction [36–38]. In this study, the Cystatin C protein was found to be significantly upregulated in both Yang deficiency and Qi-yin deficiency patients. However, like CRP protein, Cystatin C also showed a higher induced level in Yang deficiency (upregulated by 2.3-fold) than in Qi-yin deficiency (upregulated by 1.4-fold) patients.

Insulin-like growth factor binding protein 2 (IGFBP2) is a member of the IGFBP family which is present in the heart. Recent studies demonstrated the diagnostic and prognostic potential of IGFBP2 in the field of HF nonredundant with BNP [39–41]. IGFBP2 was identified to be upregulated in this study and showed an induced level of 13.9- and 4.1-fold in Yang deficiency and Qi-yin deficiency patients, respectively. The IGFBP2 level could predict the mortality risk of HF, and the high upregulated level in Yang deficiency patients may suggest that these patients have an advanced stage of HF.

Besides these HF related biomarker proteins, several disease related proteins also showed a different level of upregulation between Yang deficiency and Qi-yin deficiency patients, such as serum amyloid A-2 (SAA2, upregulated 41-fold and 17-fold, respectively) and Myosin-15 (MYH15, upregulated 9-fold and 5-fold, respectively). These results indicated that Yang deficiency patients had a more severe condition than Qi-yin deficiency patients, which is consistent with the clinical diagnosis syndromes [42].

The identification of proteins with a difference in level of induction between the two types of TCM HF syndromes could facilitate the clinical diagnosis and treatment. Adiponectin is the major insulin sensitizing adipokine, mainly secreted by hepatocytes and adipocytes. Previous studies demonstrated that adiponectin exhibits anti-inflammatory, antihyperglycemic, and antiatherogenic properties [43]. In addition, studies have also shown that adiponectin directly affects signaling in cardiac cells and is beneficial in the setting of pathological cardiac remodeling and acute cardiac injury [44]. We found that ADIPOQ was upregulated 3.26 times in Yang deficiency samples, while no significant change was detected in Qi-yin samples compared with the healthy controls. Thus, we suggest that ADIPOQ could be used as a biomarker to differentiate between the Yang and Qi-yin syndromes in clinical diagnosis.

In addition, we found that MBL2 was significantly upregulated in Qi-yin deficiency patients (upregulated by 2.26-fold) but significantly downregulated in Yang deficiency patients (downregulated to 0.27 compared to healthy control). MBL is a pattern recognition molecule of the innate immune system. Due to the important role of innate immune in producing an inflammatory response during microbial infection and tissue regeneration, MBL could be used as a potential biomarker for many human diseases [45]. Furthermore, we found that MBL2 was localized at a hub position in the PPI network; thus we suggest that MBL2 could also be used as a potential biomarker for differentiating Yang deficiency and Qi-yin deficiency syndromes.

The present study has several limitations. First, the sample size was limited with a total of 12 Yang deficiency patients, 12 Qi-yin deficiency patients, and 12 healthy controls. Although the potential biomarkers found in this study have been reported in other studies, more rigorous researches to study their specific functions and to clinically validate their sensitivity and specificity need to be conducted in another large cohort. Second, BNP, the most widely used biomarker in clinical diagnosis, was not detected in our proteomic analysis. This could be due to two reasons; one is that BNP has a very short half-life, which made it undetectable [46], and another is that some biological information in plasma could have been lost as a result of sample preparation or chromatographic gradients [47]. Third, only patients of Chinese ethnicity were enrolled due to the study design and location. Therefore, the results of this study cannot be generalized and may need replication in a cohort of patients of other ethnicities.

## 5. Conclusion

The DEPs, discovered by DIA-based MS and bioinformatics analyses, have shown biological differences between the Yang deficiency and Qi-yin deficiency TCM syndromes of HF. According to the PPI network and different expression levels, ADIPOQ, MBL2, and IGFBP4 were identified as potential biomarkers for syndrome differentiation. These results present a biological insight for diagnosing and differentiating between different TCM syndromes of HF on a scientific basis.

## Figures and Tables

**Table 1:** Clinical data of participants.

	Yang deficiency (*n* = 12)	Qi-yin deficiency (*n* = 12)	Control (*n* = 12)	*P* value
Gender (female/male)	5/7	7/5	6/6	>0.05
Age (Mean ± SD)	59−85 (71.75 ± 7.45)	54−85 (76.58 ± 9.91)	55−80 (67.75 ± 7.18)	>0.05
Hemoglobin g/L (Mean ± SD)	60−144 (108.25 ± 26.13)	108−146 (127.83 ± 10.60)	116−154 (136.09 ± 10.33)	0.002^*∗*^
Blood platelet 10^9/L (Mean ± SD)	104−325 (169.5 ± 65.37)	111−246 (174.58 ± 38.59)	123−331 (228.09 ± 68.28)	0.043^*∗*^
Alanine transaminase IU/L (Mean ± SD)	3.9–66.5 (23.31 ± 16.38)	6.2–46.5 (27.77 ± 12.57)	9.1–62.9 (26.71 ± 17.99)	0.078
Aspartate transaminase IU/L (Mean ± SD)	17.4–58.2 (35.17 ± 12.90)	17.7–56.4 (36.76 ± 10.26)	17.1–46.1 (29.77 ± 8.52)	0.284
Creatinine *μ*mol/L (Mean ± SD)	81–379.5 (192.82 ± 88.04)	50.6–105.6 (80.36 ± 16.84)	50–98.9 (67.2 ± 14.79)	<0.001^*∗*^
Na + mEq/L (Mean ± SD)	134–141.5 (136.86 ± 4.03)	131.5–141.2 (138.76 ± 3.29)	139.9–143 (141.25 ± 1.85)	0.006^*∗*^
Ka + mmol/L (Mean ± SD)	3.31–6.41 (4.38 ± 0.84)	3.26–4.88 (3.96 ± 0.44)	3.32–4.75 (4.15 ± 0.40)	0.254
CRP mg/L (Mean ± SD)	0.8–117.3 (25.44 ± 33.11)	3–50.44 (13.33 ± 17.55)	1–7.28 (2.92 ± 1.79)	0.139
BNP pg/mL (Mean ± SD)	686−31049 (13893 ± 11542)	136–28049 (7487 ± 10221)	21–102 (35.44 ± 32.37)	0.008^*∗*^

## Data Availability

The data that support the findings of this study are available from the corresponding author upon reasonable request.

## Supplementary Materials

Supplementary Materials S1 file: upregulated DEPs in Yang deficiency samples. S2 file: downregulated DEPs in Yang deficiency samples. S3 file: upregulated DEPs in Qi-yin deficiency samples. S4 file: downregulated DEPs in Qi-yin deficiency samples.
