# Supplementary material for: Identification and Comparison of Potential Biomarkers by Proteomic Analysis in Traditional Chinese Medicine-Based Heart Failure Syndromes
Source: Evid Based Complement Alternat Med. 2022 Jan 18;2022:6338508. doi: 10.1155/2022/6338508 (PMC8789435; doi:10.1155/2022/6338508)
Supplement: Supplementary Materials — S1 file: upregulated DEPs in Yang deficiency samples. S2 file: downregulated DEPs in Yang deficiency samples. S3 file: upregulated DEPs in Qi-yin deficiency samples. S4 file: downregulated DEPs in Qi-yin deficiency samples. [file 6338508.f1.zip › 6338508.f1/S1 file Up-regulated DEPs in Yang deficiency samples.docx]

| # | Protein ID | Protein group | PG_C score | PG p-value | Description | Ratio Yang VS healthy controls | p-value Yang VS healthy controls |
| --- | --- | --- | --- | --- | --- | --- | --- |
| 1 | sp\|P18428\|LBP_HUMAN | sp\|P18428\|LBP_HUMAN;tr\|Q8TCF0\|Q8TCF0_HUMAN | 9.8E-01 | 1.3E-03 | Lipopolysaccharide-binding protein OS=Homo sapiens OX=9606 GN=LBP PE=1 SV=3 | 2.687 | 0.028 |
| 2 | sp\|P62805\|H4_HUMAN | sp\|P62805\|H4_HUMAN;tr\|Q0VAS5\|Q0VAS5_HUMAN | 1.1E+00 | 1.7E-47 | Histone H4 OS=Homo sapiens OX=9606 GN=HIST1H4A PE=1 SV=2 | 7.854 | 0.002 |
| 3 | sp\|A0M8Q6\|IGLC7_HUMAN | sp\|A0M8Q6\|IGLC7_HUMAN;tr\|A0A5H1ZRQ7\|A0A5H1ZRQ7_HUMAN | 1.1E+00 | 5.0E-49 | Immunoglobulin lambda constant 7 OS=Homo sapiens OX=9606 GN=IGLC7 PE=1 SV=3 | 3.210 | 0.004 |
| 4 | sp\|P10451\|OSTP_HUMAN | sp\|P10451\|OSTP_HUMAN;tr\|A0A024RDE6\|A0A024RDE6_HUMAN;tr\|A0A024RDJ0\|A0A024RDJ0_HUMAN;tr\|B7Z351\|B7Z351_HUMAN;tr\|F2YQ21\|F2YQ21_HUMAN;tr\|Q3LGB0\|Q3LGB0_HUMAN | 1.1E+00 | 1.8E-18 | Osteopontin OS=Homo sapiens OX=9606 GN=SPP1 PE=1 SV=1 | 5.641 | ≤0.001 |
| 5 | sp\|P02741\|CRP_HUMAN | sp\|P02741\|CRP_HUMAN | 1.1E+00 | 6.6E-58 | C-reactive protein OS=Homo sapiens OX=9606 GN=CRP PE=1 SV=1 | 15.836 | ≤0.001 |
| 6 | sp\|P12724\|ECP_HUMAN | sp\|P12724\|ECP_HUMAN;tr\|W0UUR6\|W0UUR6_HUMAN | 1.1E+00 | 1.3E-28 | Eosinophil cationic protein OS=Homo sapiens OX=9606 GN=RNASE3 PE=1 SV=2 | 2.618 | 0.034 |
| 7 | tr\|Q13707\|Q13707_HUMAN | tr\|Q13707\|Q13707_HUMAN | 1.1E+00 | 1.9E-19 | ACTA2 protein (Fragment) OS=Homo sapiens OX=9606 GN=ACTA2 PE=3 SV=1 | 2.226 | 0.005 |
| 8 | sp\|P0CG47\|UBB_HUMAN | sp\|P0CG47\|UBB_HUMAN;sp\|P0CG48\|UBC_HUMAN;sp\|P62979\|RS27A_HUMAN;sp\|P62987\|RL40_HUMAN;tr\|A0A248RGE3\|A0A248RGE3_HUMAN;tr\|A8K674\|A8K674_HUMAN;tr\|B4DV12\|B4DV12_HUMAN;tr\|F5GXK7\|F5GXK7_HUMAN;tr\|F5GYU3\|F5GYU3_HUMAN;tr\|F5H265\|F5H265_HUMAN;tr\|F5H2Z3\|F5H2Z3_HUMAN;tr\|F5H388\|F5H388_HUMAN;tr\|F5H6Q2\|F5H6Q2_HUMAN;tr\|F5H747\|F5H747_HUMAN;tr\|J3QKN0\|J3QKN0_HUMAN;tr\|J3QS39\|J3QS39_HUMAN;tr\|J3QTR3\|J3QTR3_HUMAN;tr\|L8B196\|L8B196_HUMAN;tr\|L8B4I8\|L8B4I8_HUMAN;tr\|L8B4J3\|L8B4J3_HUMAN;tr\|L8B4M0\|L8B4M0_HUMAN;tr\|L8B4R0\|L8B4R0_HUMAN;tr\|L8B4Z6\|L8B4Z6_HUMAN;tr\|Q59EM9\|Q59EM9_HUMAN;tr\|Q5PY61\|Q5PY61_HUMAN;tr\|Q5RKT7\|Q5RKT7_HUMAN;tr\|Q5UGI3\|Q5UGI3_HUMAN;tr\|Q66K58\|Q66K58_HUMAN;tr\|Q96C32\|Q96C32_HUMAN;tr\|Q96H31\|Q96H31_HUMAN;tr\|Q96MH4\|Q96MH4_HUMAN;tr\|Q9UFQ0\|Q9UFQ0_HUMAN | 1.1E+00 | 2.1E-46 | Polyubiquitin-B OS=Homo sapiens OX=9606 GN=UBB PE=1 SV=1 | 2.129 | 0.013 |
| 9 | sp\|Q9NPY3\|C1QR1_HUMAN | sp\|Q9NPY3\|C1QR1_HUMAN;tr\|B4DSX2\|B4DSX2_HUMAN;tr\|Q59EB6\|Q59EB6_HUMAN;tr\|Q8IXK1\|Q8IXK1_HUMAN | 1.0E+00 | 2.1E-16 | Complement component C1q receptor OS=Homo sapiens OX=9606 GN=CD93 PE=1 SV=3 | 2.122 | 0.026 |
| 10 | sp\|O75594\|PGRP1_HUMAN | sp\|O75594\|PGRP1_HUMAN | 1.1E+00 | 3.6E-42 | Peptidoglycan recognition protein 1 OS=Homo sapiens OX=9606 GN=PGLYRP1 PE=1 SV=1 | 2.525 | 0.021 |
| 11 | sp\|P10412\|H14_HUMAN | sp\|P10412\|H14_HUMAN;tr\|A3R0T7\|A3R0T7_HUMAN;tr\|B2R984\|B2R984_HUMAN;tr\|Q4VB24\|Q4VB24_HUMAN | 1.1E+00 | 2.7E-47 | Histone H1.4 OS=Homo sapiens OX=9606 GN=HIST1H1E PE=1 SV=2 | 4.077 | 0.002 |
| 12 | sp\|P10599\|THIO_HUMAN | sp\|P10599\|THIO_HUMAN | 1.1E+00 | 2.4E-35 | Thioredoxin OS=Homo sapiens OX=9606 GN=TXN PE=1 SV=3 | 3.020 | ≤0.001 |
| 13 | tr\|B2R6W1\|B2R6W1_HUMAN | tr\|B2R6W1\|B2R6W1_HUMAN | 1.1E+00 | 3.5E-42 | cDNA, FLJ93143, highly similar to Homo sapiens complement component 7 (C7), mRNA OS=Homo sapiens OX=9606 PE=2 SV=1 | 2.166 | 0.010 |
| 14 | sp\|P40763\|STAT3_HUMAN | sp\|P40763\|STAT3_HUMAN;tr\|B4DNP0\|B4DNP0_HUMAN;tr\|B4DVR6\|B4DVR6_HUMAN;tr\|B5BTZ6\|B5BTZ6_HUMAN;tr\|B7ZA24\|B7ZA24_HUMAN;tr\|G8JLH9\|G8JLH9_HUMAN | 9.6E-01 | 7.0E-03 | Signal transducer and activator of transcription 3 OS=Homo sapiens OX=9606 GN=STAT3 PE=1 SV=2 | 5.230 | 0.012 |
| 15 | sp\|Q9H299\|SH3L3_HUMAN | sp\|Q9H299\|SH3L3_HUMAN;tr\|D3DPK5\|D3DPK5_HUMAN;tr\|Q5T123\|Q5T123_HUMAN;tr\|Q86Z22\|Q86Z22_HUMAN | 1.1E+00 | 2.4E-34 | SH3 domain-binding glutamic acid-rich-like protein 3 OS=Homo sapiens OX=9606 GN=SH3BGRL3 PE=1 SV=1 | 2.841 | ≤0.001 |
| 16 | sp\|Q4KMP7\|TB10B_HUMAN | sp\|Q4KMP7\|TB10B_HUMAN | 9.9E-01 | 3.7E-05 | TBC1 domain family member 10B OS=Homo sapiens OX=9606 GN=TBC1D10B PE=1 SV=3 | 3.468 | 0.012 |
| 17 | sp\|P67936\|TPM4_HUMAN | sp\|P67936\|TPM4_HUMAN;tr\|A0A2R8Y5V9\|A0A2R8Y5V9_HUMAN;tr\|B4DVY2\|B4DVY2_HUMAN | 1.1E+00 | 1.4E-46 | Tropomyosin alpha-4 chain OS=Homo sapiens OX=9606 GN=TPM4 PE=1 SV=3 | 5.310 | 0.019 |
| 18 | tr\|Q68CK4\|Q68CK4_HUMAN | tr\|Q68CK4\|Q68CK4_HUMAN | 1.1E+00 | 3.5E-38 | Leucine-rich alpha-2-glycoprotein OS=Homo sapiens OX=9606 GN=HMFT1766 PE=2 SV=1 | 3.719 | 0.326 |
| 19 | sp\|P07988\|PSPB_HUMAN | sp\|P07988\|PSPB_HUMAN;tr\|D6W5L6\|D6W5L6_HUMAN | 1.1E+00 | 5.9E-46 | Pulmonary surfactant-associated protein B OS=Homo sapiens OX=9606 GN=SFTPB PE=1 SV=3 | 5.145 | 0.006 |
| 20 | sp\|P02763\|A1AG1_HUMAN | sp\|P02763\|A1AG1_HUMAN | 1.1E+00 | 1.9E-54 | Alpha-1-acid glycoprotein 1 OS=Homo sapiens OX=9606 GN=ORM1 PE=1 SV=1 | 2.290 | 0.004 |
| 21 | tr\|Q6MZX7\|Q6MZX7_HUMAN | tr\|Q6MZX7\|Q6MZX7_HUMAN | 1.1E+00 | 1.4E-47 | Uncharacterized protein DKFZp686M24218 OS=Homo sapiens OX=9606 GN=DKFZp686M24218 PE=2 SV=1 | 1.019 | 0.938 |
| 22 | tr\|A0A1L7B5J3\|A0A1L7B5J3_HUMAN | tr\|A0A1L7B5J3\|A0A1L7B5J3_HUMAN | 1.1E+00 | 1.2E-40 | Alpha-1-antitrypsin short transcript variant 1C4 OS=Homo sapiens OX=9606 GN=SERPINA1 PE=2 SV=1 | 2.356 | 0.002 |
| 23 | sp\|P09382\|LEG1_HUMAN | sp\|P09382\|LEG1_HUMAN | 1.1E+00 | 9.3E-45 | Galectin-1 OS=Homo sapiens OX=9606 GN=LGALS1 PE=1 SV=2 | 2.521 | ≤0.001 |
| 24 | sp\|P37802\|TAGL2_HUMAN | sp\|P37802\|TAGL2_HUMAN;tr\|X6RJP6\|X6RJP6_HUMAN | 1.1E+00 | 4.9E-53 | Transgelin-2 OS=Homo sapiens OX=9606 GN=TAGLN2 PE=1 SV=3 | 4.697 | ≤0.001 |
| 25 | sp\|P05109\|S10A8_HUMAN | sp\|P05109\|S10A8_HUMAN | 1.1E+00 | 7.1E-56 | Protein S100-A8 OS=Homo sapiens OX=9606 GN=S100A8 PE=1 SV=1 | 3.340 | 0.005 |
| 26 | sp\|P63104\|1433Z_HUMAN | sp\|P63104\|1433Z_HUMAN;tr\|E5RGE1\|E5RGE1_HUMAN;tr\|E5RIR4\|E5RIR4_HUMAN;tr\|E7ESK7\|E7ESK7_HUMAN;tr\|E7EVZ2\|E7EVZ2_HUMAN;tr\|E7EX29\|E7EX29_HUMAN;tr\|E9PD24\|E9PD24_HUMAN;tr\|Q6LD62\|Q6LD62_HUMAN | 1.1E+00 | 1.2E-23 | 14-3-3 protein zeta/delta OS=Homo sapiens OX=9606 GN=YWHAZ PE=1 SV=1 | 2.450 | 0.005 |
| 27 | sp\|P62328\|TYB4_HUMAN | sp\|P62328\|TYB4_HUMAN | 1.1E+00 | 5.1E-58 | Thymosin beta-4 OS=Homo sapiens OX=9606 GN=TMSB4X PE=1 SV=2 | 3.320 | ≤0.001 |
| 28 | sp\|P08311\|CATG_HUMAN | sp\|P08311\|CATG_HUMAN | 1.1E+00 | 1.1E-31 | Cathepsin G OS=Homo sapiens OX=9606 GN=CTSG PE=1 SV=2 | 2.282 | 0.029 |
| 29 | tr\|B3KTL6\|B3KTL6_HUMAN | tr\|B3KTL6\|B3KTL6_HUMAN | 1.0E+00 | 7.4E-07 | cDNA FLJ38470 fis, clone FEBRA2022013, highly similar to CCR4-NOT transcription complex subunit 2 (Fragment) OS=Homo sapiens OX=9606 PE=2 SV=1 | 2.096 | 0.027 |
| 30 | sp\|O00151\|PDLI1_HUMAN | sp\|O00151\|PDLI1_HUMAN | 1.1E+00 | 8.7E-44 | PDZ and LIM domain protein 1 OS=Homo sapiens OX=9606 GN=PDLIM1 PE=1 SV=4 | 4.560 | ≤0.001 |
| 31 | sp\|P16401\|H15_HUMAN | sp\|P16401\|H15_HUMAN | 1.1E+00 | 1.1E-41 | Histone H1.5 OS=Homo sapiens OX=9606 GN=HIST1H1B PE=1 SV=3 | 5.828 | 0.004 |
| 32 | sp\|Q16819\|MEP1A_HUMAN | sp\|Q16819\|MEP1A_HUMAN;tr\|B7ZL91\|B7ZL91_HUMAN | 1.0E+00 | 1.2E-05 | Meprin A subunit alpha OS=Homo sapiens OX=9606 GN=MEP1A PE=1 SV=2 | 4.114 | 0.016 |
| 33 | sp\|P36222\|CH3L1_HUMAN | sp\|P36222\|CH3L1_HUMAN | 1.1E+00 | 1.9E-47 | Chitinase-3-like protein 1 OS=Homo sapiens OX=9606 GN=CHI3L1 PE=1 SV=2 | 9.836 | 0.002 |
| 34 | sp\|Q14697\|GANAB_HUMAN | sp\|Q14697\|GANAB_HUMAN;tr\|B4DJ30\|B4DJ30_HUMAN | 1.1E+00 | 4.4E-25 | Neutral alpha-glucosidase AB OS=Homo sapiens OX=9606 GN=GANAB PE=1 SV=3 | 2.632 | 0.009 |
| 35 | sp\|P01034\|CYTC_HUMAN | sp\|P01034\|CYTC_HUMAN | 1.1E+00 | 1.2E-58 | Cystatin-C OS=Homo sapiens OX=9606 GN=CST3 PE=1 SV=1 | 2.344 | 0.002 |
| 36 | sp\|Q3ZCW2\|LEGL_HUMAN | sp\|Q3ZCW2\|LEGL_HUMAN | 1.1E+00 | 2.3E-38 | Galectin-related protein OS=Homo sapiens OX=9606 GN=LGALSL PE=1 SV=2 | 2.893 | 0.009 |
| 37 | tr\|H0YLF3\|H0YLF3_HUMAN | tr\|H0YLF3\|H0YLF3_HUMAN | 1.1E+00 | 1.3E-43 | Beta-2-microglobulin (Fragment) OS=Homo sapiens OX=9606 GN=B2M PE=1 SV=1 | 0.875 | 0.846 |
| 38 | sp\|P04908\|H2A1B_HUMAN | sp\|P04908\|H2A1B_HUMAN;sp\|P0C0S8\|H2A1_HUMAN;sp\|P20671\|H2A1D_HUMAN;sp\|Q16777\|H2A2C_HUMAN;sp\|Q6FI13\|H2A2A_HUMAN;sp\|Q7L7L0\|H2A3_HUMAN;sp\|Q93077\|H2A1C_HUMAN;sp\|Q96KK5\|H2A1H_HUMAN;sp\|Q99878\|H2A1J_HUMAN;sp\|Q9BTM1\|H2AJ_HUMAN;tr\|A0A0U1RR32\|A0A0U1RR32_HUMAN;tr\|A0A0U1RRH7\|A0A0U1RRH7_HUMAN;tr\|B2R5B3\|B2R5B3_HUMAN;tr\|B4E0B3\|B4E0B3_HUMAN;tr\|H0YFX9\|H0YFX9_HUMAN | 1.1E+00 | 1.8E-39 | Histone H2A type 1-B/E OS=Homo sapiens OX=9606 GN=HIST1H2AB PE=1 SV=2 | 4.368 | 0.009 |
| 39 | tr\|Q9Y509\|Q9Y509_HUMAN | tr\|Q9Y509\|Q9Y509_HUMAN | 1.1E+00 | 2.4E-60 | VH3 protein (Fragment) OS=Homo sapiens OX=9606 GN=VH3 PE=2 SV=1 | 1.184 | 0.515 |
| 40 | sp\|P61224\|RAP1B_HUMAN | sp\|P61224\|RAP1B_HUMAN | 1.0E+00 | 4.9E-06 | Ras-related protein Rap-1b OS=Homo sapiens OX=9606 GN=RAP1B PE=1 SV=1 | 2.547 | 0.015 |
| 41 | sp\|Q9Y2K3\|MYH15_HUMAN | sp\|Q9Y2K3\|MYH15_HUMAN;tr\|B3KP05\|B3KP05_HUMAN | 1.1E+00 | 5.6E-27 | Myosin-15 OS=Homo sapiens OX=9606 GN=MYH15 PE=1 SV=5 | 9.150 | 0.009 |
| 42 | sp\|P60709\|ACTB_HUMAN | sp\|P60709\|ACTB_HUMAN;tr\|Q53G76\|Q53G76_HUMAN;tr\|Q53G99\|Q53G99_HUMAN;tr\|Q53GK6\|Q53GK6_HUMAN | 1.1E+00 | 4.8E-52 | Actin, cytoplasmic 1 OS=Homo sapiens OX=9606 GN=ACTB PE=1 SV=1 | 2.717 | ≤0.001 |
| 43 | sp\|Q9P1F3\|ABRAL_HUMAN | sp\|Q9P1F3\|ABRAL_HUMAN | 1.1E+00 | 3.8E-38 | Costars family protein ABRACL OS=Homo sapiens OX=9606 GN=ABRACL PE=1 SV=1 | 3.119 | 0.022 |
| 44 | sp\|P62942\|FKB1A_HUMAN | sp\|P62942\|FKB1A_HUMAN | 1.1E+00 | 2.3E-24 | Peptidyl-prolyl cis-trans isomerase FKBP1A OS=Homo sapiens OX=9606 GN=FKBP1A PE=1 SV=2 | 2.101 | 0.023 |
| 45 | sp\|Q13790\|APOF_HUMAN | sp\|Q13790\|APOF_HUMAN;tr\|B2RC09\|B2RC09_HUMAN | 1.1E+00 | 1.5E-46 | Apolipoprotein F OS=Homo sapiens OX=9606 GN=APOF PE=1 SV=2 | 3.128 | ≤0.001 |
| 46 | sp\|O60814\|H2B1K_HUMAN | sp\|O60814\|H2B1K_HUMAN;sp\|P57053\|H2BFS_HUMAN;sp\|P58876\|H2B1D_HUMAN;sp\|P62807\|H2B1C_HUMAN;sp\|Q5QNW6\|H2B2F_HUMAN;sp\|Q93079\|H2B1H_HUMAN;sp\|Q99877\|H2B1N_HUMAN;sp\|Q99879\|H2B1M_HUMAN;sp\|Q99880\|H2B1L_HUMAN;tr\|A8K9J7\|A8K9J7_HUMAN;tr\|B4DR52\|B4DR52_HUMAN;tr\|I6L9F7\|I6L9F7_HUMAN;tr\|Q0D2M2\|Q0D2M2_HUMAN;tr\|U3KQK0\|U3KQK0_HUMAN | 1.1E+00 | 3.5E-42 | Histone H2B type 1-K OS=Homo sapiens OX=9606 GN=HIST1H2BK PE=1 SV=3 | 2.876 | ≤0.001 |
| 47 | sp\|P27348\|1433T_HUMAN | sp\|P27348\|1433T_HUMAN;tr\|E9PG15\|E9PG15_HUMAN;tr\|Q53RR5\|Q53RR5_HUMAN | 1.1E+00 | 8.4E-31 | 14-3-3 protein theta OS=Homo sapiens OX=9606 GN=YWHAQ PE=1 SV=1 | 2.404 | 0.005 |
| 48 | sp\|P02144\|MYG_HUMAN | sp\|P02144\|MYG_HUMAN;tr\|A0A024R1G3\|A0A024R1G3_HUMAN;tr\|B0QYF8\|B0QYF8_HUMAN | 1.1E+00 | 3.4E-45 | Myoglobin OS=Homo sapiens OX=9606 GN=MB PE=1 SV=2 | 3.683 | 0.013 |
| 49 | sp\|P13987\|CD59_HUMAN | sp\|P13987\|CD59_HUMAN;tr\|A0A2U3TZL5\|A0A2U3TZL5_HUMAN;tr\|E9PNW4\|E9PNW4_HUMAN | 1.1E+00 | 3.6E-34 | CD59 glycoprotein OS=Homo sapiens OX=9606 GN=CD59 PE=1 SV=1 | 3.796 | ≤0.001 |
| 50 | sp\|P04080\|CYTB_HUMAN | sp\|P04080\|CYTB_HUMAN | 1.1E+00 | 5.4E-19 | Cystatin-B OS=Homo sapiens OX=9606 GN=CSTB PE=1 SV=2 | 3.481 | ≤0.001 |
| 51 | sp\|P22692\|IBP4_HUMAN | sp\|P22692\|IBP4_HUMAN | 1.1E+00 | 4.3E-45 | Insulin-like growth factor-binding protein 4 OS=Homo sapiens OX=9606 GN=IGFBP4 PE=1 SV=2 | 3.000 | ≤0.001 |
| 52 | sp\|P07737\|PROF1_HUMAN | sp\|P07737\|PROF1_HUMAN | 1.1E+00 | 2.0E-43 | Profilin-1 OS=Homo sapiens OX=9606 GN=PFN1 PE=1 SV=2 | 3.222 | ≤0.001 |
| 53 | sp\|P62937\|PPIA_HUMAN | sp\|P62937\|PPIA_HUMAN;tr\|A8K486\|A8K486_HUMAN | 1.1E+00 | 4.7E-45 | Peptidyl-prolyl cis-trans isomerase A OS=Homo sapiens OX=9606 GN=PPIA PE=1 SV=2 | 2.325 | 0.003 |
| 54 | tr\|D3DQX7\|D3DQX7_HUMAN | tr\|D3DQX7\|D3DQX7_HUMAN | 1.1E+00 | 1.2E-57 | Serum amyloid A protein OS=Homo sapiens OX=9606 GN=SAA1 PE=3 SV=1 | 52.590 | 0.003 |
| 55 | sp\|Q92520\|FAM3C_HUMAN | sp\|Q92520\|FAM3C_HUMAN | 1.1E+00 | 1.8E-19 | Protein FAM3C OS=Homo sapiens OX=9606 GN=FAM3C PE=1 SV=1 | 2.488 | 0.008 |
| 56 | sp\|P80188\|NGAL_HUMAN | sp\|P80188\|NGAL_HUMAN;tr\|B2ZDQ1\|B2ZDQ1_HUMAN;tr\|X6R8F3\|X6R8F3_HUMAN | 1.1E+00 | 5.1E-35 | Neutrophil gelatinase-associated lipocalin OS=Homo sapiens OX=9606 GN=LCN2 PE=1 SV=2 | 2.192 | 0.005 |
| 57 | sp\|P05451\|REG1A_HUMAN | sp\|P05451\|REG1A_HUMAN;sp\|P48304\|REG1B_HUMAN;tr\|A8K7G6\|A8K7G6_HUMAN | 1.1E+00 | 9.8E-26 | Lithostathine-1-alpha OS=Homo sapiens OX=9606 GN=REG1A PE=1 SV=3 | 2.973 | ≤0.001 |
| 58 | sp\|P0DJI9\|SAA2_HUMAN | sp\|P0DJI9\|SAA2_HUMAN | 1.1E+00 | 8.2E-57 | Serum amyloid A-2 protein OS=Homo sapiens OX=9606 GN=SAA2 PE=1 SV=1 | 41.693 | 0.004 |
| 59 | sp\|P50281\|MMP14_HUMAN | sp\|P50281\|MMP14_HUMAN;tr\|B2R6P3\|B2R6P3_HUMAN;tr\|F8W1B7\|F8W1B7_HUMAN;tr\|K4RH61\|K4RH61_HUMAN | 1.1E+00 | 5.1E-44 | Matrix metalloproteinase-14 OS=Homo sapiens OX=9606 GN=MMP14 PE=1 SV=3 | 2.496 | 0.003 |
| 60 | tr\|A0A0U4EXB4\|A0A0U4EXB4_HUMAN | tr\|A0A0U4EXB4\|A0A0U4EXB4_HUMAN | 1.1E+00 | 2.2E-51 | Haptoglobin (Fragment) OS=Homo sapiens OX=9606 GN=HP PE=4 SV=1 | 3.273 | 0.002 |
| 61 | sp\|P09603\|CSF1_HUMAN | sp\|P09603\|CSF1_HUMAN;tr\|A0A024R0A1\|A0A024R0A1_HUMAN;tr\|B4DTX0\|B4DTX0_HUMAN | 1.1E+00 | 4.8E-35 | Macrophage colony-stimulating factor 1 OS=Homo sapiens OX=9606 GN=CSF1 PE=1 SV=2 | 2.739 | ≤0.001 |
| 62 | sp\|Q14508\|WFDC2_HUMAN | sp\|Q14508\|WFDC2_HUMAN | 1.1E+00 | 2.3E-36 | WAP four-disulfide core domain protein 2 OS=Homo sapiens OX=9606 GN=WFDC2 PE=1 SV=2 | 7.120 | 0.006 |
| 63 | sp\|P06702\|S10A9_HUMAN | sp\|P06702\|S10A9_HUMAN | 1.1E+00 | 1.9E-56 | Protein S100-A9 OS=Homo sapiens OX=9606 GN=S100A9 PE=1 SV=1 | 3.258 | 0.004 |
| 64 | tr\|Q9UL86\|Q9UL86_HUMAN | tr\|Q9UL86\|Q9UL86_HUMAN | 1.1E+00 | 7.7E-50 | Myosin-reactive immunoglobulin kappa chain variable region (Fragment) OS=Homo sapiens OX=9606 PE=2 SV=1 | 0.791 | 0.315 |
| 65 | sp\|P16403\|H12_HUMAN | sp\|P16403\|H12_HUMAN | 1.1E+00 | 2.3E-34 | Histone H1.2 OS=Homo sapiens OX=9606 GN=HIST1H1C PE=1 SV=2 | 3.317 | 0.006 |
| 66 | sp\|P07998\|RNAS1_HUMAN | sp\|P07998\|RNAS1_HUMAN | 1.1E+00 | 6.4E-42 | Ribonuclease pancreatic OS=Homo sapiens OX=9606 GN=RNASE1 PE=1 SV=4 | 2.973 | 0.004 |
| 67 | tr\|A8K5T0\|A8K5T0_HUMAN | tr\|A8K5T0\|A8K5T0_HUMAN | 1.1E+00 | 7.9E-50 | cDNA FLJ75416, highly similar to Homo sapiens complement factor H (CFH), mRNA OS=Homo sapiens OX=9606 PE=2 SV=1 | 0.800 | 0.350 |
| 68 | sp\|Q15828\|CYTM_HUMAN | sp\|Q15828\|CYTM_HUMAN;tr\|Q6IBD2\|Q6IBD2_HUMAN | 1.1E+00 | 6.8E-36 | Cystatin-M OS=Homo sapiens OX=9606 GN=CST6 PE=1 SV=1 | 2.021 | 0.017 |
| 69 | sp\|P18065\|IBP2_HUMAN | sp\|P18065\|IBP2_HUMAN | 1.1E+00 | 4.3E-30 | Insulin-like growth factor-binding protein 2 OS=Homo sapiens OX=9606 GN=IGFBP2 PE=1 SV=2 | 13.946 | ≤0.001 |
| 70 | sp\|P41222\|PTGDS_HUMAN | sp\|P41222\|PTGDS_HUMAN | 1.1E+00 | 2.7E-44 | Prostaglandin-H2 D-isomerase OS=Homo sapiens OX=9606 GN=PTGDS PE=1 SV=1 | 2.277 | ≤0.001 |
| 71 | sp\|P08493\|MGP_HUMAN | sp\|P08493\|MGP_HUMAN | 1.1E+00 | 3.1E-58 | Matrix Gla protein OS=Homo sapiens OX=9606 GN=MGP PE=1 SV=2 | 2.216 | 0.004 |
| 72 | sp\|P61626\|LYSC_HUMAN | sp\|P61626\|LYSC_HUMAN | 1.1E+00 | 8.6E-54 | Lysozyme C OS=Homo sapiens OX=9606 GN=LYZ PE=1 SV=1 | 2.851 | 0.002 |
| 73 | sp\|Q15848\|ADIPO_HUMAN | sp\|Q15848\|ADIPO_HUMAN;tr\|A0A3B0J0F2\|A0A3B0J0F2_HUMAN;tr\|B2R773\|B2R773_HUMAN | 1.1E+00 | 2.5E-38 | Adiponectin OS=Homo sapiens OX=9606 GN=ADIPOQ PE=1 SV=1 | 3.269 | 0.021 |
| 74 | sp\|Q8WWA1\|TMM40_HUMAN | sp\|Q8WWA1\|TMM40_HUMAN | 1.1E+00 | 6.1E-50 | Transmembrane protein 40 OS=Homo sapiens OX=9606 GN=TMEM40 PE=1 SV=2 | 7.043 | ≤0.001 |
| 75 | sp\|P05062\|ALDOB_HUMAN | sp\|P05062\|ALDOB_HUMAN;tr\|A0A087WXX2\|A0A087WXX2_HUMAN;tr\|A0A3B3IS80\|A0A3B3IS80_HUMAN;tr\|A0A3B3ITZ0\|A0A3B3ITZ0_HUMAN;tr\|A8K430\|A8K430_HUMAN;tr\|Q8NHT3\|Q8NHT3_HUMAN | 9.7E-01 | 4.3E-03 | Fructose-bisphosphate aldolase B OS=Homo sapiens OX=9606 GN=ALDOB PE=1 SV=2 | 3.900 | 0.010 |
| 76 | sp\|P30046\|DOPD_HUMAN | sp\|P30046\|DOPD_HUMAN;tr\|J3KQ18\|J3KQ18_HUMAN | 1.0E+00 | 6.4E-17 | D-dopachrome decarboxylase OS=Homo sapiens OX=9606 GN=DDT PE=1 SV=3 | 2.641 | 0.023 |
| 77 | sp\|Q14767\|LTBP2_HUMAN | sp\|Q14767\|LTBP2_HUMAN;tr\|G3V3X5\|G3V3X5_HUMAN;tr\|G3V511\|G3V511_HUMAN;tr\|Q6AZ94\|Q6AZ94_HUMAN | 1.1E+00 | 2.8E-40 | Latent-transforming growth factor beta-binding protein 2 OS=Homo sapiens OX=9606 GN=LTBP2 PE=1 SV=3 | 2.023 | 0.030 |
