# Supplementary material for: Identification and Comparison of Potential Biomarkers by Proteomic Analysis in Traditional Chinese Medicine-Based Heart Failure Syndromes
Source: Evid Based Complement Alternat Med. 2022 Jan 18;2022:6338508. doi: 10.1155/2022/6338508 (PMC8789435; doi:10.1155/2022/6338508)
Supplement: Supplementary Materials — S1 file: upregulated DEPs in Yang deficiency samples. S2 file: downregulated DEPs in Yang deficiency samples. S3 file: upregulated DEPs in Qi-yin deficiency samples. S4 file: downregulated DEPs in Qi-yin deficiency samples. [file 6338508.f1.zip › 6338508.f1/S2 file Down-regulated DEPs in Yang deficiency samples.docx]

| # | Protein ID | Protein group | PG_C score | PG P-value | Description | Ratio Yang VS healthy controls | P-value Yang VS healthy controls |
| --- | --- | --- | --- | --- | --- | --- | --- |
| 1 | sp\|P02549\|SPTA1_HUMAN | sp\|P02549\|SPTA1_HUMAN;tr\|D3DVD8\|D3DVD8_HUMAN | 1.094128 | 8.55E-38 | Spectrin alpha chain, erythrocytic 1 OS=Homo sapiens OX=9606 GN=SPTA1 PE=1 SV=5 | 0.095 | 0.014 |
| 2 | sp\|Q8NFZ3\|NLGNY_HUMAN | sp\|Q8NFZ3\|NLGNY_HUMAN;tr\|A6NMU8\|A6NMU8_HUMAN;tr\|B4DHI3\|B4DHI3_HUMAN | 1.088173 | 1.07E-34 | Neuroligin-4, Y-linked OS=Homo sapiens OX=9606 GN=NLGN4Y PE=2 SV=1 | 0.246 | 0.012 |
| 3 | sp\|Q14623\|IHH_HUMAN | sp\|Q14623\|IHH_HUMAN;tr\|Q4ZFW8\|Q4ZFW8_HUMAN | 1.109623 | 1.78E-46 | Indian hedgehog protein OS=Homo sapiens OX=9606 GN=IHH PE=1 SV=4 | 0.487 | 0.003 |
| 4 | tr\|A0A1U9WZ84\|A0A1U9WZ84_HUMAN | tr\|A0A1U9WZ84\|A0A1U9WZ84_HUMAN | 1.084583 | 6.90E-33 | Insulin-like growth factor I (Fragment) OS=Homo sapiens OX=9606 PE=3 SV=1 | 0.284 | 0.032 |
| 5 | sp\|Q14520\|HABP2_HUMAN | sp\|Q14520\|HABP2_HUMAN | 1.10919 | 3.16E-46 | Hyaluronan-binding protein 2 OS=Homo sapiens OX=9606 GN=HABP2 PE=1 SV=1 | 0.165 | ≤0.001 |
| 6 | sp\|O96006\|ZBED1_HUMAN | sp\|O96006\|ZBED1_HUMAN | 1.11112 | 2.36E-47 | Zinc finger BED domain-containing protein 1 OS=Homo sapiens OX=9606 GN=ZBED1 PE=1 SV=1 | 0.323 | ≤0.001 |
| 7 | sp\|P02776\|PLF4_HUMAN | sp\|P02776\|PLF4_HUMAN | 1.121786 | 8.66E-54 | Platelet factor 4 OS=Homo sapiens OX=9606 GN=PF4 PE=1 SV=2 | 0.381 | 0.002 |
| 8 | sp\|P08697\|A2AP_HUMAN | sp\|P08697\|A2AP_HUMAN | 1.116654 | 1.18E-50 | Alpha-2-antiplasmin OS=Homo sapiens OX=9606 GN=SERPINF2 PE=1 SV=3 | 0.453 | 0.002 |
| 9 | tr\|K7ER74\|K7ER74_HUMAN | tr\|K7ER74\|K7ER74_HUMAN | 1.129767 | 3.12E-58 | APOC4-APOC2 readthrough (NMD candidate) OS=Homo sapiens OX=9606 GN=APOC4-APOC2 PE=1 SV=1 | 0.360 | 0.002 |
| 10 | sp\|Q92954\|PRG4_HUMAN | sp\|Q92954\|PRG4_HUMAN;tr\|A0A024R930\|A0A024R930_HUMAN;tr\|B3KQ20\|B3KQ20_HUMAN | 1.130346 | 1.65E-58 | Proteoglycan 4 OS=Homo sapiens OX=9606 GN=PRG4 PE=1 SV=3 | 0.383 | ≤0.001 |
| 11 | sp\|Q86U17\|SPA11_HUMAN | sp\|Q86U17\|SPA11_HUMAN | 1.053363 | 1.93E-19 | Serpin A11 OS=Homo sapiens OX=9606 GN=SERPINA11 PE=2 SV=2 | 0.347 | 0.028 |
| 12 | sp\|A0A075B6S6\|KVD30_HUMAN | sp\|A0A075B6S6\|KVD30_HUMAN;tr\|A0A5C2GUY0\|A0A5C2GUY0_HUMAN | 1.070978 | 1.52E-26 | Immunoglobulin kappa variable 2D-30 OS=Homo sapiens OX=9606 GN=IGKV2D-30 PE=3 SV=1 | 1.010 | 0.974 |
| 13 | sp\|P43652\|AFAM_HUMAN | sp\|P43652\|AFAM_HUMAN | 1.120066 | 9.64E-53 | Afamin OS=Homo sapiens OX=9606 GN=AFM PE=1 SV=1 | 0.485 | ≤0.001 |
| 14 | sp\|Q15154\|PCM1_HUMAN | sp\|Q15154\|PCM1_HUMAN;tr\|A0A4W8VX11\|A0A4W8VX11_HUMAN;tr\|A0A5H1ZRS1\|A0A5H1ZRS1_HUMAN;tr\|A2RUU9\|A2RUU9_HUMAN;tr\|B9EIS5\|B9EIS5_HUMAN;tr\|D3DSQ0\|D3DSQ0_HUMAN | 1.045844 | 8.32E-17 | Pericentriolar material 1 protein OS=Homo sapiens OX=9606 GN=PCM1 PE=1 SV=5 | 0.462 | ≤0.001 |
| 15 | sp\|A0A1B0GTC6\|CC085_HUMAN | sp\|A0A1B0GTC6\|CC085_HUMAN;tr\|A0A1B0GVL1\|A0A1B0GVL1_HUMAN;tr\|A0A1B0GVS5\|A0A1B0GVS5_HUMAN | 1.060928 | 2.47E-22 | Uncharacterized protein C3orf85 OS=Homo sapiens OX=9606 GN=C3orf85 PE=3 SV=1 | 0.347 | 0.037 |
| 16 | tr\|A5PL32\|A5PL32_HUMAN | tr\|A5PL32\|A5PL32_HUMAN;tr\|Q1WWL5\|Q1WWL5_HUMAN;tr\|Q29RW7\|Q29RW7_HUMAN;tr\|Q2KHQ6\|Q2KHQ6_HUMAN | 0.991914 | 8.62E-05 | APOL1 protein (Fragment) OS=Homo sapiens OX=9606 GN=APOL1 PE=2 SV=1 | - | - |
| 17 | tr\|Q68DR3\|Q68DR3_HUMAN | tr\|Q68DR3\|Q68DR3_HUMAN | 1.084695 | 6.07E-33 | Uncharacterized protein DKFZp779H1622 (Fragment) OS=Homo sapiens OX=9606 GN=DKFZp779H1622 PE=2 SV=1 | 0.240 | ≤0.001 |
| 18 | sp\|P11226\|MBL2_HUMAN | sp\|P11226\|MBL2_HUMAN | 1.121245 | 1.82E-53 | Mannose-binding protein C OS=Homo sapiens OX=9606 GN=MBL2 PE=1 SV=2 | 0.276 | 0.009 |
| 19 | tr\|Q9Y509\|Q9Y509_HUMAN | tr\|Q9Y509\|Q9Y509_HUMAN | 1.13442 | 2.38E-60 | VH3 protein (Fragment) OS=Homo sapiens OX=9606 GN=VH3 PE=2 SV=1 | 1.184 | 0.515 |
| 20 | tr\|B2R582\|B2R582_HUMAN | tr\|B2R582\|B2R582_HUMAN | 1.095862 | 9.97E-39 | cDNA, FLJ92374, highly similar to Homo sapiens C-type lectin domain family 3, member B (CLEC3B), mRNA OS=Homo sapiens OX=9606 PE=2 SV=1 | 0.388 | 0.002 |
| 21 | sp\|P05154\|IPSP_HUMAN | sp\|P05154\|IPSP_HUMAN | 1.110639 | 4.49E-47 | Plasma serine protease inhibitor OS=Homo sapiens OX=9606 GN=SERPINA5 PE=1 SV=3 | 0.299 | ≤0.001 |
| 22 | tr\|V9GYG9\|V9GYG9_HUMAN | tr\|V9GYG9\|V9GYG9_HUMAN | 1.134879 | 1.68E-60 | Apolipoprotein A-II (Fragment) OS=Homo sapiens OX=9606 GN=APOA2 PE=1 SV=1 | 0.462 | 0.002 |
| 23 | sp\|P53618\|COPB_HUMAN | sp\|P53618\|COPB_HUMAN | 1.053815 | 1.31E-19 | Coatomer subunit beta OS=Homo sapiens OX=9606 GN=COPB1 PE=1 SV=3 | 0.328 | 0.026 |
| 24 | sp\|P55056\|APOC4_HUMAN | sp\|P55056\|APOC4_HUMAN;tr\|A5YAK2\|A5YAK2_HUMAN | 1.11954 | 2.06E-52 | Apolipoprotein C-IV OS=Homo sapiens OX=9606 GN=APOC4 PE=1 SV=1 | 0.149 | ≤0.001 |
| 25 | sp\|P19827\|ITIH1_HUMAN | sp\|P19827\|ITIH1_HUMAN | 1.113803 | 6.08E-49 | Inter-alpha-trypsin inhibitor heavy chain H1 OS=Homo sapiens OX=9606 GN=ITIH1 PE=1 SV=3 | 0.433 | 0.005 |
| 26 | sp\|P02765\|FETUA_HUMAN | sp\|P02765\|FETUA_HUMAN;tr\|B7Z8Q2\|B7Z8Q2_HUMAN | 1.119542 | 2.06E-52 | Alpha-2-HS-glycoprotein OS=Homo sapiens OX=9606 GN=AHSG PE=1 SV=2 | 0.466 | 0.004 |
| 27 | tr\|V9HW68\|V9HW68_HUMAN | tr\|V9HW68\|V9HW68_HUMAN | 1.130121 | 2.12E-58 | Epididymis luminal protein 214 OS=Homo sapiens OX=9606 GN=HEL-214 PE=2 SV=1 | 1.006 | 0.976 |
| 28 | tr\|A2J1N1\|A2J1N1_HUMAN | tr\|A2J1N1\|A2J1N1_HUMAN | 1.001894 | 4.09E-06 | Rheumatoid factor RF-IP15 (Fragment) OS=Homo sapiens OX=9606 PE=2 SV=1 | 1.662 | 0.153 |
| 29 | tr\|Q86TT2\|Q86TT2_HUMAN | tr\|Q86TT2\|Q86TT2_HUMAN | 1.125198 | 7.87E-56 | Full-length cDNA clone CS0DI019YF20 of Placenta of Homo sapiens (human) (Fragment) OS=Homo sapiens OX=9606 PE=2 SV=1 | 0.396 | 0.044 |
| 30 | sp\|P35858\|ALS_HUMAN | sp\|P35858\|ALS_HUMAN;tr\|Q8TAY0\|Q8TAY0_HUMAN | 1.129263 | 4.99E-58 | Insulin-like growth factor-binding protein complex acid labile subunit OS=Homo sapiens OX=9606 GN=IGFALS PE=1 SV=1 | 0.310 | ≤0.001 |
| 31 | sp\|P04180\|LCAT_HUMAN | sp\|P04180\|LCAT_HUMAN | 1.121985 | 6.56E-54 | Phosphatidylcholine-sterol acyltransferase OS=Homo sapiens OX=9606 GN=LCAT PE=1 SV=1 | 0.421 | 0.004 |
| 32 | sp\|P26927\|HGFL_HUMAN | sp\|P26927\|HGFL_HUMAN;tr\|G3XAK1\|G3XAK1_HUMAN;tr\|Q53GN8\|Q53GN8_HUMAN | 1.097888 | 7.99E-40 | Hepatocyte growth factor-like protein OS=Homo sapiens OX=9606 GN=MST1 PE=1 SV=2 | 0.429 | 0.005 |
| 33 | sp\|Q9HCJ2\|LRC4C_HUMAN | sp\|Q9HCJ2\|LRC4C_HUMAN;tr\|Q4JIV9\|Q4JIV9_HUMAN;tr\|Q4JIW0\|Q4JIW0_HUMAN | 1.06094 | 2.45E-22 | Leucine-rich repeat-containing protein 4C OS=Homo sapiens OX=9606 GN=LRRC4C PE=1 SV=1 | 0.424 | 0.005 |
| 34 | tr\|Q9NPP6\|Q9NPP6_HUMAN | tr\|Q9NPP6\|Q9NPP6_HUMAN | 1.125301 | 7.07E-56 | Immunoglobulin heavy chain variant (Fragment) OS=Homo sapiens OX=9606 PE=2 SV=1 | 1.606 | 0.109 |
| 35 | sp\|P60985\|KTDAP_HUMAN | sp\|P60985\|KTDAP_HUMAN | 1.076115 | 7.43E-29 | Keratinocyte differentiation-associated protein OS=Homo sapiens OX=9606 GN=KRTDAP PE=1 SV=1 | 0.217 | ≤0.001 |
| 36 | tr\|S6C4R7\|S6C4R7_HUMAN | tr\|S6C4R7\|S6C4R7_HUMAN | 1.112155 | 5.97E-48 | IgG L chain OS=Homo sapiens OX=9606 PE=2 SV=1 | 0.587 | 0.301 |
| 37 | sp\|O75636\|FCN3_HUMAN | sp\|O75636\|FCN3_HUMAN | 1.118181 | 1.41E-51 | Ficolin-3 OS=Homo sapiens OX=9606 GN=FCN3 PE=1 SV=2 | 0.390 | ≤0.001 |
| 38 | sp\|Q6DHV5\|C2D2B_HUMAN | sp\|Q6DHV5\|C2D2B_HUMAN | 1.111529 | 1.36E-47 | Protein CC2D2B OS=Homo sapiens OX=9606 GN=CC2D2B PE=2 SV=3 | 0.463 | 0.002 |
| 39 | tr\|A2N0U4\|A2N0U4_HUMAN | tr\|A2N0U4\|A2N0U4_HUMAN | 1.053218 | 2.17E-19 | VH6DJ protein (Fragment) OS=Homo sapiens OX=9606 GN=VH6DJ PE=2 SV=1 | 1.974 | 0.278 |
| 40 | tr\|S6B2C3\|S6B2C3_HUMAN | tr\|S6B2C3\|S6B2C3_HUMAN | 1.084095 | 1.19E-32 | IgG L chain OS=Homo sapiens OX=9606 PE=2 SV=1 | 0.365 | 0.031 |
| 41 | tr\|Q5NV82\|Q5NV82_HUMAN | tr\|Q5NV82\|Q5NV82_HUMAN | 1.105118 | 7.23E-44 | V4-2 protein (Fragment) OS=Homo sapiens OX=9606 GN=V4-2 PE=4 SV=1 | 0.593 | 0.091 |
| 42 | tr\|A2IPI6\|A2IPI6_HUMAN | tr\|A2IPI6\|A2IPI6_HUMAN | 1.104993 | 8.51E-44 | HRV Fab 027-VL (Fragment) OS=Homo sapiens OX=9606 PE=2 SV=1 | 0.883 | 0.612 |
| 43 | sp\|P02654\|APOC1_HUMAN | sp\|P02654\|APOC1_HUMAN;tr\|K7ERI9\|K7ERI9_HUMAN | 1.12336 | 1.01E-54 | Apolipoprotein C-I OS=Homo sapiens OX=9606 GN=APOC1 PE=1 SV=1 | 0.402 | 0.021 |
| 44 | sp\|P05546\|HEP2_HUMAN | sp\|P05546\|HEP2_HUMAN | 1.115292 | 7.77E-50 | Heparin cofactor 2 OS=Homo sapiens OX=9606 GN=SERPIND1 PE=1 SV=3 | 0.354 | 0.024 |
