# Supplementary material for: Identification and Comparison of Potential Biomarkers by Proteomic Analysis in Traditional Chinese Medicine-Based Heart Failure Syndromes
Source: Evid Based Complement Alternat Med. 2022 Jan 18;2022:6338508. doi: 10.1155/2022/6338508 (PMC8789435; doi:10.1155/2022/6338508)
Supplement: Supplementary Materials — S1 file: upregulated DEPs in Yang deficiency samples. S2 file: downregulated DEPs in Yang deficiency samples. S3 file: upregulated DEPs in Qi-yin deficiency samples. S4 file: downregulated DEPs in Qi-yin deficiency samples. [file 6338508.f1.zip › 6338508.f1/S3 file Up-regulated DEPs in Qi-yin deficiency samples.docx]

| # | Protein ID | Protein group | PG_C score | PG P-value | Description | Ratio Yang VS healthy controls | P-value Yang VS healthy controls |
| --- | --- | --- | --- | --- | --- | --- | --- |
| 1 | sp\|P62805\|H4_HUMAN | sp\|P62805\|H4_HUMAN;tr\|Q0VAS5\|Q0VAS5_HUMAN | 1.111346 | 1.73E-47 | Histone H4 OS=Homo sapiens OX=9606 GN=HIST1H4A PE=1 SV=2 | 3.634 | 0.022 |
| 2 | sp\|P10451\|OSTP_HUMAN | sp\|P10451\|OSTP_HUMAN;tr\|A0A024RDE6\|A0A024RDE6_HUMAN;tr\|A0A024RDJ0\|A0A024RDJ0_HUMAN;tr\|B7Z351\|B7Z351_HUMAN;tr\|F2YQ21\|F2YQ21_HUMAN;tr\|Q3LGB0\|Q3LGB0_HUMAN | 1.050693 | 1.77E-18 | Osteopontin OS=Homo sapiens OX=9606 GN=SPP1 PE=1 SV=1 | 2.446 | 0.003 |
| 3 | sp\|P02741\|CRP_HUMAN | sp\|P02741\|CRP_HUMAN | 1.128936 | 6.56E-58 | C-reactive protein OS=Homo sapiens OX=9606 GN=CRP PE=1 SV=1 | 8.604 | 0.005 |
| 4 | sp\|P12724\|ECP_HUMAN | sp\|P12724\|ECP_HUMAN;tr\|W0UUR6\|W0UUR6_HUMAN | 1.075541 | 1.34E-28 | Eosinophil cationic protein OS=Homo sapiens OX=9606 GN=RNASE3 PE=1 SV=2 | 4.163 | 0.005 |
| 5 | sp\|P02748\|CO9_HUMAN | sp\|P02748\|CO9_HUMAN;tr\|A0A024R035\|A0A024R035_HUMAN | 1.10562 | 3.73E-44 | Complement component C9 OS=Homo sapiens OX=9606 GN=C9 PE=1 SV=2 | 2.126 | 0.004 |
| 6 | sp\|O75594\|PGRP1_HUMAN | sp\|O75594\|PGRP1_HUMAN | 1.102138 | 3.56E-42 | Peptidoglycan recognition protein 1 OS=Homo sapiens OX=9606 GN=PGLYRP1 PE=1 SV=1 | 2.263 | 0.037 |
| 7 | sp\|P10412\|H14_HUMAN | sp\|P10412\|H14_HUMAN;tr\|A3R0T7\|A3R0T7_HUMAN;tr\|B2R984\|B2R984_HUMAN;tr\|Q4VB24\|Q4VB24_HUMAN | 1.111012 | 2.68E-47 | Histone H1.4 OS=Homo sapiens OX=9606 GN=HIST1H1E PE=1 SV=2 | 4.006 | 0.002 |
| 8 | sp\|P10599\|THIO_HUMAN | sp\|P10599\|THIO_HUMAN | 1.089455 | 2.40E-35 | Thioredoxin OS=Homo sapiens OX=9606 GN=TXN PE=1 SV=3 | 2.262 | 0.002 |
| 9 | tr\|Q9UL82\|Q9UL82_HUMAN | tr\|Q9UL82\|Q9UL82_HUMAN | 1.10366 | 4.90E-43 | Myosin-reactive immunoglobulin light chain variable region (Fragment) OS=Homo sapiens OX=9606 PE=2 SV=1 | 2.538 | 0.058 |
| 10 | sp\|P40763\|STAT3_HUMAN | sp\|P40763\|STAT3_HUMAN;tr\|B4DNP0\|B4DNP0_HUMAN;tr\|B4DVR6\|B4DVR6_HUMAN;tr\|B5BTZ6\|B5BTZ6_HUMAN;tr\|B7ZA24\|B7ZA24_HUMAN;tr\|G8JLH9\|G8JLH9_HUMAN | 0.9552055 | 0.007004028 | Signal transducer and activator of transcription 3 OS=Homo sapiens OX=9606 GN=STAT3 PE=1 SV=2 | 3.297 | 0.045 |
| 11 | sp\|Q9H299\|SH3L3_HUMAN | sp\|Q9H299\|SH3L3_HUMAN;tr\|D3DPK5\|D3DPK5_HUMAN;tr\|Q5T123\|Q5T123_HUMAN;tr\|Q86Z22\|Q86Z22_HUMAN | 1.087498 | 2.37E-34 | SH3 domain-binding glutamic acid-rich-like protein 3 OS=Homo sapiens OX=9606 GN=SH3BGRL3 PE=1 SV=1 | 3.060 | ≤0.001 |
| 12 | sp\|P59665\|DEF1_HUMAN | sp\|P59665\|DEF1_HUMAN;sp\|P59666\|DEF3_HUMAN | 1.113804 | 6.08E-49 | Neutrophil defensin 1 OS=Homo sapiens OX=9606 GN=DEFA1 PE=1 SV=1 | 2.766 | 0.002 |
| 13 | sp\|Q4KMP7\|TB10B_HUMAN | sp\|Q4KMP7\|TB10B_HUMAN | 0.9949495 | 3.75E-05 | TBC1 domain family member 10B OS=Homo sapiens OX=9606 GN=TBC1D10B PE=1 SV=3 | 3.977 | 0.009 |
| 14 | sp\|P67936\|TPM4_HUMAN | sp\|P67936\|TPM4_HUMAN;tr\|A0A2R8Y5V9\|A0A2R8Y5V9_HUMAN;tr\|B4DVY2\|B4DVY2_HUMAN | 1.109781 | 1.45E-46 | Tropomyosin alpha-4 chain OS=Homo sapiens OX=9606 GN=TPM4 PE=1 SV=3 | 5.159 | 0.021 |
| 15 | sp\|P07988\|PSPB_HUMAN | sp\|P07988\|PSPB_HUMAN;tr\|D6W5L6\|D6W5L6_HUMAN | 1.108714 | 5.93E-46 | Pulmonary surfactant-associated protein B OS=Homo sapiens OX=9606 GN=SFTPB PE=1 SV=3 | 3.550 | 0.021 |
| 16 | sp\|Q96QR1\|SG3A1_HUMAN | sp\|Q96QR1\|SG3A1_HUMAN | 1.10018 | 4.46E-41 | Secretoglobin family 3A member 1 OS=Homo sapiens OX=9606 GN=SCGB3A1 PE=1 SV=2 | 2.038 | 0.033 |
| 17 | sp\|P37802\|TAGL2_HUMAN | sp\|P37802\|TAGL2_HUMAN;tr\|X6RJP6\|X6RJP6_HUMAN | 1.120554 | 4.90E-53 | Transgelin-2 OS=Homo sapiens OX=9606 GN=TAGLN2 PE=1 SV=3 | 4.619 | ≤0.001 |
| 18 | sp\|P05109\|S10A8_HUMAN | sp\|P05109\|S10A8_HUMAN | 1.125306 | 7.07E-56 | Protein S100-A8 OS=Homo sapiens OX=9606 GN=S100A8 PE=1 SV=1 | 2.346 | 0.028 |
| 19 | sp\|P62328\|TYB4_HUMAN | sp\|P62328\|TYB4_HUMAN | 1.129218 | 5.07E-58 | Thymosin beta-4 OS=Homo sapiens OX=9606 GN=TMSB4X PE=1 SV=2 | 3.121 | ≤0.001 |
| 20 | sp\|P08311\|CATG_HUMAN | sp\|P08311\|CATG_HUMAN | 1.082139 | 1.07E-31 | Cathepsin G OS=Homo sapiens OX=9606 GN=CTSG PE=1 SV=2 | 2.386 | 0.016 |
| 21 | sp\|O00151\|PDLI1_HUMAN | sp\|O00151\|PDLI1_HUMAN | 1.104972 | 8.72E-44 | PDZ and LIM domain protein 1 OS=Homo sapiens OX=9606 GN=PDLIM1 PE=1 SV=4 | 4.382 | ≤0.001 |
| 22 | sp\|P16401\|H15_HUMAN | sp\|P16401\|H15_HUMAN | 1.101276 | 1.09E-41 | Histone H1.5 OS=Homo sapiens OX=9606 GN=HIST1H1B PE=1 SV=3 | 5.792 | 0.004 |
| 23 | sp\|P36222\|CH3L1_HUMAN | sp\|P36222\|CH3L1_HUMAN | 1.111282 | 1.88E-47 | Chitinase-3-like protein 1 OS=Homo sapiens OX=9606 GN=CHI3L1 PE=1 SV=2 | 4.434 | 0.018 |
| 24 | sp\|Q14697\|GANAB_HUMAN | sp\|Q14697\|GANAB_HUMAN;tr\|B4DJ30\|B4DJ30_HUMAN | 1.067608 | 4.36E-25 | Neutral alpha-glucosidase AB OS=Homo sapiens OX=9606 GN=GANAB PE=1 SV=3 | 2.394 | 0.019 |
| 25 | sp\|Q3ZCW2\|LEGL_HUMAN | sp\|Q3ZCW2\|LEGL_HUMAN | 1.095177 | 2.34E-38 | Galectin-related protein OS=Homo sapiens OX=9606 GN=LGALSL PE=1 SV=2 | 2.875 | 0.010 |
| 26 | sp\|Q0VAA2\|LR74A_HUMAN | sp\|Q0VAA2\|LR74A_HUMAN;tr\|B4DX10\|B4DX10_HUMAN;tr\|G3V356\|G3V356_HUMAN;tr\|H7BXD0\|H7BXD0_HUMAN;tr\|Q9Y628\|Q9Y628_HUMAN | 1.103374 | 7.10E-43 | Leucine-rich repeat-containing protein 74A OS=Homo sapiens OX=9606 GN=LRRC74A PE=2 SV=2 | 2.320 | 0.017 |
| 27 | tr\|H0YLF3\|H0YLF3_HUMAN | tr\|H0YLF3\|H0YLF3_HUMAN | 1.104676 | 1.30E-43 | Beta-2-microglobulin (Fragment) OS=Homo sapiens OX=9606 GN=B2M PE=1 SV=1 | 1.936 | 0.348 |
| 28 | sp\|P04908\|H2A1B_HUMAN | sp\|P04908\|H2A1B_HUMAN;sp\|P0C0S8\|H2A1_HUMAN;sp\|P20671\|H2A1D_HUMAN;sp\|Q16777\|H2A2C_HUMAN;sp\|Q6FI13\|H2A2A_HUMAN;sp\|Q7L7L0\|H2A3_HUMAN;sp\|Q93077\|H2A1C_HUMAN;sp\|Q96KK5\|H2A1H_HUMAN;sp\|Q99878\|H2A1J_HUMAN;sp\|Q9BTM1\|H2AJ_HUMAN;tr\|A0A0U1RR32\|A0A0U1RR32_HUMAN;tr\|A0A0U1RRH7\|A0A0U1RRH7_HUMAN;tr\|B2R5B3\|B2R5B3_HUMAN;tr\|B4E0B3\|B4E0B3_HUMAN;tr\|H0YFX9\|H0YFX9_HUMAN | 1.097221 | 1.83E-39 | Histone H2A type 1-B/E OS=Homo sapiens OX=9606 GN=HIST1H2AB PE=1 SV=2 | 6.370 | 0.002 |
| 29 | sp\|Q9Y2K3\|MYH15_HUMAN | sp\|Q9Y2K3\|MYH15_HUMAN;tr\|B3KP05\|B3KP05_HUMAN | 1.071957 | 5.58E-27 | Myosin-15 OS=Homo sapiens OX=9606 GN=MYH15 PE=1 SV=5 | 5.278 | 0.033 |
| 30 | sp\|P60709\|ACTB_HUMAN | sp\|P60709\|ACTB_HUMAN;tr\|Q53G76\|Q53G76_HUMAN;tr\|Q53G99\|Q53G99_HUMAN;tr\|Q53GK6\|Q53GK6_HUMAN | 1.118935 | 4.83E-52 | Actin, cytoplasmic 1 OS=Homo sapiens OX=9606 GN=ACTB PE=1 SV=1 | 2.245 | ≤0.001 |
| 31 | sp\|Q9P1F3\|ABRAL_HUMAN | sp\|Q9P1F3\|ABRAL_HUMAN | 1.094778 | 3.82E-38 | Costars family protein ABRACL OS=Homo sapiens OX=9606 GN=ABRACL PE=1 SV=1 | 2.736 | 0.037 |
| 32 | sp\|O60814\|H2B1K_HUMAN | sp\|O60814\|H2B1K_HUMAN;sp\|P57053\|H2BFS_HUMAN;sp\|P58876\|H2B1D_HUMAN;sp\|P62807\|H2B1C_HUMAN;sp\|Q5QNW6\|H2B2F_HUMAN;sp\|Q93079\|H2B1H_HUMAN;sp\|Q99877\|H2B1N_HUMAN;sp\|Q99879\|H2B1M_HUMAN;sp\|Q99880\|H2B1L_HUMAN;tr\|A8K9J7\|A8K9J7_HUMAN;tr\|B4DR52\|B4DR52_HUMAN;tr\|I6L9F7\|I6L9F7_HUMAN;tr\|Q0D2M2\|Q0D2M2_HUMAN;tr\|U3KQK0\|U3KQK0_HUMAN | 1.10215 | 3.52E-42 | Histone H2B type 1-K OS=Homo sapiens OX=9606 GN=HIST1H2BK PE=1 SV=3 | 5.054 | ≤0.001 |
| 33 | tr\|Q86TT2\|Q86TT2_HUMAN | tr\|Q86TT2\|Q86TT2_HUMAN | 1.125198 | 7.87E-56 | Full-length cDNA clone CS0DI019YF20 of Placenta of Homo sapiens (human) (Fragment) OS=Homo sapiens OX=9606 PE=2 SV=1 | 2.481 | 0.047 |
| 34 | sp\|P20160\|CAP7_HUMAN | sp\|P20160\|CAP7_HUMAN;tr\|Q86SR2\|Q86SR2_HUMAN | 1.055513 | 3.07E-20 | Azurocidin OS=Homo sapiens OX=9606 GN=AZU1 PE=1 SV=3 | 3.392 | 0.044 |
| 35 | sp\|P13987\|CD59_HUMAN | sp\|P13987\|CD59_HUMAN;tr\|A0A2U3TZL5\|A0A2U3TZL5_HUMAN;tr\|E9PNW4\|E9PNW4_HUMAN | 1.087147 | 3.56E-34 | CD59 glycoprotein OS=Homo sapiens OX=9606 GN=CD59 PE=1 SV=1 | 2.710 | ≤0.001 |
| 36 | sp\|P07737\|PROF1_HUMAN | sp\|P07737\|PROF1_HUMAN | 1.104361 | 1.96E-43 | Profilin-1 OS=Homo sapiens OX=9606 GN=PFN1 PE=1 SV=2 | 3.856 | ≤0.001 |
| 37 | sp\|P62937\|PPIA_HUMAN | sp\|P62937\|PPIA_HUMAN;tr\|A8K486\|A8K486_HUMAN | 1.107167 | 4.71E-45 | Peptidyl-prolyl cis-trans isomerase A OS=Homo sapiens OX=9606 GN=PPIA PE=1 SV=2 | 2.337 | 0.003 |
| 38 | tr\|D3DQX7\|D3DQX7_HUMAN | tr\|D3DQX7\|D3DQX7_HUMAN | 1.128417 | 1.21E-57 | Serum amyloid A protein OS=Homo sapiens OX=9606 GN=SAA1 PE=3 SV=1 | 16.557 | 0.017 |
| 39 | sp\|P80188\|NGAL_HUMAN | sp\|P80188\|NGAL_HUMAN;tr\|B2ZDQ1\|B2ZDQ1_HUMAN;tr\|X6R8F3\|X6R8F3_HUMAN | 1.088813 | 5.09E-35 | Neutrophil gelatinase-associated lipocalin OS=Homo sapiens OX=9606 GN=LCN2 PE=1 SV=2 | 2.361 | 0.003 |
| 40 | sp\|P05451\|REG1A_HUMAN | sp\|P05451\|REG1A_HUMAN;sp\|P48304\|REG1B_HUMAN;tr\|A8K7G6\|A8K7G6_HUMAN | 1.069122 | 9.76E-26 | Lithostathine-1-alpha OS=Homo sapiens OX=9606 GN=REG1A PE=1 SV=3 | 2.076 | 0.014 |
| 41 | sp\|P0DJI9\|SAA2_HUMAN | sp\|P0DJI9\|SAA2_HUMAN | 1.126925 | 8.15E-57 | Serum amyloid A-2 protein OS=Homo sapiens OX=9606 GN=SAA2 PE=1 SV=1 | 17.009 | 0.016 |
| 42 | sp\|P31146\|COR1A_HUMAN | sp\|P31146\|COR1A_HUMAN;tr\|Q59G88\|Q59G88_HUMAN | 1.079354 | 2.30E-30 | Coronin-1A OS=Homo sapiens OX=9606 GN=CORO1A PE=1 SV=4 | 3.576 | 0.020 |
| 43 | sp\|Q14508\|WFDC2_HUMAN | sp\|Q14508\|WFDC2_HUMAN | 1.091423 | 2.29E-36 | WAP four-disulfide core domain protein 2 OS=Homo sapiens OX=9606 GN=WFDC2 PE=1 SV=2 | 3.554 | 0.038 |
| 44 | sp\|P06702\|S10A9_HUMAN | sp\|P06702\|S10A9_HUMAN | 1.126291 | 1.90E-56 | Protein S100-A9 OS=Homo sapiens OX=9606 GN=S100A9 PE=1 SV=1 | 2.756 | 0.009 |
| 45 | sp\|P16403\|H12_HUMAN | sp\|P16403\|H12_HUMAN | 1.087507 | 2.35E-34 | Histone H1.2 OS=Homo sapiens OX=9606 GN=HIST1H1C PE=1 SV=2 | 3.300 | 0.007 |
| 46 | sp\|P18065\|IBP2_HUMAN | sp\|P18065\|IBP2_HUMAN | 1.078783 | 4.28E-30 | Insulin-like growth factor-binding protein 2 OS=Homo sapiens OX=9606 GN=IGFBP2 PE=1 SV=2 | 4.145 | 0.024 |
| 47 | sp\|P61626\|LYSC_HUMAN | sp\|P61626\|LYSC_HUMAN | 1.121796 | 8.62E-54 | Lysozyme C OS=Homo sapiens OX=9606 GN=LYZ PE=1 SV=1 | 2.347 | 0.007 |
| 48 | sp\|Q8WWA1\|TMM40_HUMAN | sp\|Q8WWA1\|TMM40_HUMAN | 1.115482 | 6.12E-50 | Transmembrane protein 40 OS=Homo sapiens OX=9606 GN=TMEM40 PE=1 SV=2 | 6.310 | ≤0.001 |
| 49 | sp\|P05062\|ALDOB_HUMAN | sp\|P05062\|ALDOB_HUMAN;tr\|A0A087WXX2\|A0A087WXX2_HUMAN;tr\|A0A3B3IS80\|A0A3B3IS80_HUMAN;tr\|A0A3B3ITZ0\|A0A3B3ITZ0_HUMAN;tr\|A8K430\|A8K430_HUMAN;tr\|Q8NHT3\|Q8NHT3_HUMAN | 0.9673231 | 0.004340895 | Fructose-bisphosphate aldolase B OS=Homo sapiens OX=9606 GN=ALDOB PE=1 SV=2 | 3.143 | 0.013 |
