# Supplementary material for: Identification and Comparison of Potential Biomarkers by Proteomic Analysis in Traditional Chinese Medicine-Based Heart Failure Syndromes
Source: Evid Based Complement Alternat Med. 2022 Jan 18;2022:6338508. doi: 10.1155/2022/6338508 (PMC8789435; doi:10.1155/2022/6338508)
Supplement: Supplementary Materials — S1 file: upregulated DEPs in Yang deficiency samples. S2 file: downregulated DEPs in Yang deficiency samples. S3 file: upregulated DEPs in Qi-yin deficiency samples. S4 file: downregulated DEPs in Qi-yin deficiency samples. [file 6338508.f1.zip › 6338508.f1/S4 file Down-regulated DEPs in Qi-yin deficiency samples.docx]

| # | Protein ID | Protein group | PG_C score | PG P-value | Description | Ratio Yang VS healthy controls | P-value Yang VS healthy controls |
| --- | --- | --- | --- | --- | --- | --- | --- |
| 1 | sp\|Q8NFZ3\|NLGNY_HUMAN | sp\|Q8NFZ3\|NLGNY_HUMAN;tr\|A6NMU8\|A6NMU8_HUMAN;tr\|B4DHI3\|B4DHI3_HUMAN | 1.088173 | 1.07E-34 | Neuroligin-4, Y-linked OS=Homo sapiens OX=9606 GN=NLGN4Y PE=2 SV=1 | 0.231 | 0.010 |
| 2 | tr\|A0A1U9WZ84\|A0A1U9WZ84_HUMAN | tr\|A0A1U9WZ84\|A0A1U9WZ84_HUMAN | 1.084583 | 6.90E-33 | Insulin-like growth factor I (Fragment) OS=Homo sapiens OX=9606 PE=3 SV=1 | 0.279 | 0.021 |
| 3 | sp\|P02776\|PLF4_HUMAN | sp\|P02776\|PLF4_HUMAN | 1.121786 | 8.66E-54 | Platelet factor 4 OS=Homo sapiens OX=9606 GN=PF4 PE=1 SV=2 | 0.466 | 0.006 |
| 4 | sp\|A0A075B6S6\|KVD30_HUMAN | sp\|A0A075B6S6\|KVD30_HUMAN;tr\|A0A5C2GUY0\|A0A5C2GUY0_HUMAN | 1.070978 | 1.52E-26 | Immunoglobulin kappa variable 2D-30 OS=Homo sapiens OX=9606 GN=IGKV2D-30 PE=3 SV=1 | 1.295 | 0.423 |
| 5 | tr\|Q9Y509\|Q9Y509_HUMAN | tr\|Q9Y509\|Q9Y509_HUMAN | 1.13442 | 2.38E-60 | VH3 protein (Fragment) OS=Homo sapiens OX=9606 GN=VH3 PE=2 SV=1 | 1.139 | 0.615 |
| 6 | tr\|A2JA19\|A2JA19_HUMAN | tr\|A2JA19\|A2JA19_HUMAN | 1.105116 | 7.23E-44 | Anti-mucin1 light chain variable region (Fragment) OS=Homo sapiens OX=9606 PE=2 SV=1 | 1.742 | 0.258 |
| 7 | sp\|Q9ULV4\|COR1C_HUMAN | sp\|Q9ULV4\|COR1C_HUMAN;tr\|B3KN06\|B3KN06_HUMAN;tr\|B4DMH3\|B4DMH3_HUMAN;tr\|B4E3S0\|B4E3S0_HUMAN;tr\|B7Z9V0\|B7Z9V0_HUMAN;tr\|H0YHL7\|H0YHL7_HUMAN;tr\|Q53G58\|Q53G58_HUMAN;tr\|Q59EA2\|Q59EA2_HUMAN | 1.064633 | 7.74E-24 | Coronin-1C OS=Homo sapiens OX=9606 GN=CORO1C PE=1 SV=1 | 0.283 | 0.033 |
| 8 | sp\|P55056\|APOC4_HUMAN | sp\|P55056\|APOC4_HUMAN;tr\|A5YAK2\|A5YAK2_HUMAN | 1.11954 | 2.06E-52 | Apolipoprotein C-IV OS=Homo sapiens OX=9606 GN=APOC4 PE=1 SV=1 | 0.272 | 0.006 |
| 9 | tr\|A2N0U4\|A2N0U4_HUMAN | tr\|A2N0U4\|A2N0U4_HUMAN | 1.053218 | 2.17E-19 | VH6DJ protein (Fragment) OS=Homo sapiens OX=9606 GN=VH6DJ PE=2 SV=1 | 0.529 | 0.301 |
| 10 | sp\|P05546\|HEP2_HUMAN | sp\|P05546\|HEP2_HUMAN | 1.115292 | 7.77E-50 | Heparin cofactor 2 OS=Homo sapiens OX=9606 GN=SERPIND1 PE=1 SV=3 | 0.351 | 0.024 |
